# Genome-wide association study and transcriptomic analysis reveal the crucial role of *sting1* in resistance to visceral white-nodules disease in *Larimichthys polyactis*

**DOI:** 10.3389/fimmu.2025.1562307

**Published:** 2025-04-28

**Authors:** Jiajie Zhu, Feng Liu, Ting Ye, Qian Li, Haowen Liu, Sifang Liu, Tianle Zhang, Dandan Guo, Junquan Zhu, Bao Lou

**Affiliations:** ^1^ Key Laboratory of Applied Marine Biotechnology by the Ministry of Education, School of Marine Sciences, Ningbo University, Ningbo, China; ^2^ State Key Laboratory for Quality and Safety of Agro-Products / Institute of Hydrobiology, Zhejiang Academy of Agricultural Sciences, Hangzhou, China; ^3^ National Engineering Research Center for Marine Aquaculture, Zhejiang Ocean University, Zhoushan, China; ^4^ College of Life Sciences, China Jiliang University, Hangzhou, China

**Keywords:** Larimichthys polyactis, visceral white-nodules disease, GWAS, transcriptome, STING1

## Abstract

**Introduction:**

*Larimichthys polyactis* is a promising marine fishery species, but visceral white-nodules disease (VWND) caused by *Pseudomonas plecoglossicida* causes significant losses. However, genetic resistance mechanisms to VWND remain elusive in this species.

**Methods:**

This study combined genome-wide association study (GWAS) and transcriptome analysis to unravel resistance loci and transcriptional regulation in *L. polyactis*.

**Results:**

As a result, GWAS on 946 infected fish genotyped by 100 K lipid chips identified 22 suggestive significantly associated single-nucleotide polymorphisms (SNPs), annotated 60 candidate genes, where DNA-sensing pathway were enriched. RNA-seq on liver tissues of resistant, sensitive, and control groups found immune-related pathways enriched in the comparisons of RL vs CL and RL vs SL, and autophagy-related pathways enriched in the comparisons of SL vs CL and RL vs SL. Then, the integration of GWAS and transcriptome analysis identified seven key genes associated with resistance to VWND. Among the genes, the expression levels of mRNA for genes related to the cyclic GMP-AMP synthase-stimulator of interferon genes (STING) signaling pathway, as well as the protein levels of STING1, were significantly upregulated in RL. Collectively, integrating KEGG pathway analysis, gene and protein expression analysis revealed that the importance of STING1 for VWND resistance.

**Discussion:**

These findings deepen the available knowledge on molecular mechanisms of host genetic resistance to VWND and provide an important foundation for the selection and breeding of VWND-resistant *L. polyactis*.

## 1 Introduction

Aquaculture is increasingly contributing to global food supply and healthy nutrition, and a large number of new aquaculture farms have emerged (1). However, the associated risk is still high, partly because of infectious diseases, which have become a key constraint on the sustainability of the entire aquaculture production system. *Larimichthys polyactis* is an economically extremely promising marine fishery species that is cultured in the coastal areas of Zhejiang and Jiangsu provinces, China (2). However, high density cultivation usually results in outbreaks of many diseases. Among those, visceral white-nodules disease (VWND) caused by *Pseudomonas plecoglossicida* is one of the most prevalent and devastating diseases (3), which results in granulomas in the liver and spleen of the infected fish. It causes huge economic losses to the *L. polyactis* industry. This disease is highly infectious and insidious, and can cause mass deaths within a short period of time (7-10 days). Therefore, it is crucial to elucidate the molecular mechanisms underlying immune response regulation and to breed strains of *L. polyactis* with high resistance to VWND.

Disease resistance traits are complex and have a complex genetic basis (4); therefore, it is difficult to explain them with simple models. Genome-wide association study (GWAS) is a means of finding the genetic basis of complex diseases (5). Based on linkage disequilibrium, GWAS utilizes millions of single-nucleotide polymorphisms (SNPs) to search for genetic variants associated with specific phenotypes (6). With the completion of the sequencing of the genomes of various aquatic animals, the development of commercial chips, and advances in genome sequencing, high-density genotyping of numerous individuals has become possible. As a result, many GWAS analyses exploring genetic resistances to diseases have focused on fish, and many SNP markers and functional genes have been identified to date (7, 8). Marana et al. (9) identify 33 candidate genes associated with *Aeromonas salmonicida* resistance in *Oncorhynchus mykiss* by performing GWAS for survival and time to death phenotypes with the 57 K SNP array. In interspecific hybrid catfish (*Ictalurus punctatus* ♀ × *Ictalurus furcatus* ♂), 24 immune related genes were identified to be associated with Motile *Aeromonas septicemia* disease resistance, 10 of which were involved in NF-κB signaling pathway (10). This not only greatly enriches the development of molecular markers for disease resistance breeding, but also provides important clues for research on disease resistance mechanisms.

Understanding the genetic mechanisms underpinning disease resistance is a challenging process. An approach that integrates transcriptomic data with genomic data to identify candidate genes and pathways associated with resistance has been applied in research on aquaculture. In *Salmo salar*, one significantly associated region was identified conferring resistance to the infectious salmon anaemia virus; the interferon pathway characterized the response to the virus in the heart (11). Similarly, several relevant networks were found to mediate genetic resistance to Salmon Rickettsial Syndrome, including apoptosis, cytoskeletal organization, and the inflammasome (12). Boison et al. (13) incorporated gene expression information into the result of GWAS to improve the resistance of breeding programs to amoebic gill disease. A study showed that the p53 signaling pathway mediates VWND resistance in *Larimichthys crocea* by integrating of GWAS data and RNA-seq data (14). However, combining GWAS information and transcriptome results to elucidate disease resistance mechanisms in *L. polyactis* remains unexplored.

The aim of this study was to identify genes associated with resistance to VWND and reveal their regulatory mechanisms in *L. polyactis*. A total of 946 infected fish were genotyped by 100 K lipid chips to obtain SNP information and identify VWND resistance candidate genes. Further, RNA-seq was performed on another 27 fish (both resistant and susceptible individuals) to examine regulatory pathways against infection. The crossover method provides insights into the molecular mechanisms of VWND resistance in *L. polyactis*, as well as a theoretical basis for the molecular breeding of disease resistance traits and the development of genetic engineering.

## 2 Materials and methods

### 2.1 Fish and challenge test

The bacteria were taken from the lesioned liver and spleen of diseased fish (a significant *L. polyactis* mortality event in May 2022) by inoculation loops and cultured on tryptic soy agar (TSA) plates at 28°C for 24 h. Single colonies from plates were restreaked onto TSA plates to obtain pure colonies. All pure colonies were stored in tryptic soy broth (TSB) with 25% sterile glycerol at -80 °C. The bacterial strain was identified as *P. plecoglossicida* (unpublished). A total of 950 eight-month-old *L. polyactis* (body weight 22.72 ± 7.65 g, body length 11.05 ± 1.22 cm), obtained from the population cultured in Xiangshan harbor aquatic seedling Co. LTD (Ningbo, China), were randomly divided into 10 two-ton tanks (95 fish per tank). Fish were temporarily cultured for a two-weeks acclimation period in filtered seawater (18 °C) and were fed with an artificial compound feed.

To determine their resistance to VWND, fish were challenged with *P. plecoglossicida* by intraperitoneally injection. Feeding was stopped 24 h before the start of the experiment. The bacteria isolated above were cultured in TSB at 28°C for 24 h and then centrifugated at 3000 rpm for 3 min. The bacterial cells were washed twice and adjusted to a final concentration of 1.0 × 10^3^ colony forming unit (CFU/mL) with phosphate-buffered saline (PBS). A suitable infection dosage of 0.5 mL of 1.0 × 10^3^ CFU/mL of *P. plecoglossicida* that led to about 50% overall mortality in fish (tested at 96 h) was determined by a pre-experiment. Moreover, fish of the control group (three two-ton tanks, 95 fish per tank) were intraperitoneally injected with 0.5 mL PBS without bacteria. The challenged fish were closely observed and collected immediately when they were observed either lying motionless on the bottom of the tank or floating unbalanced on the water surface, showing no response even when touched. The time of death also known as survival time (ST), was recorded as the phenotype for resistance to VWND. After both body weight and body length were measured, fish were dissected to examine viscera symptoms, and the caudal fin was collected and stored in absolute ethyl alcohol.

Additionally, 200 fish (body weight 28.55 ± 7.73 g, body length 11.73 ± 1.08 cm) from a full-sib family were randomly divided into two two-ton tanks, and were subjected to an intraperitoneal injection challenge with the pre-determined dose (0.5 mL of 1.0 × 10^3^ CFU/mL) of *P. plecoglossicida*. Fish of the control group (CL) (three two-ton tanks, 100 fish per tank) were intraperitoneally injected with 0.5 mL sterile PBS. The challenged fish were also collected immediately when they lost equilibrium and were belly-up. The fish of CL were sampled simultaneously. Liver tissues were collected and immediately flash-frozen in liquid nitrogen until further analysis. In this experiment, the first nine sampled individuals were defined as the sensitive group (SL), and the last nine sampled individuals were recorded as the resistant group (RL). A brief schematic diagram of the experimental is shown in **Supplementary Figure S1**.

All experiments were reviewed and approved by the Committee of Laboratory Animal Experimentation at Zhejiang Academy of Agricultural Sciences (Hangzhou, China). Sampled fish were anesthetized using tricaine methanesulfonate (MS-222; Sigma, St. Louis, MO, USA).

### 2.2 DNA extraction and genotyping

Genomic DNA was extracted by a commercial kit (Omega Bio-Tek, Norcross, GA, USA), and quantified by Qubit 4.0 (Thermo Fisher Scientific, Waltham, MA, USA). DNA integrity was examined by 1.2% agarose gel electrophoresis. Acceptable samples were sent to Higentec Co., Ltd. (Changsha, Hunan, China) for genotyping with 100 K liquid SNP chips, which included 100,031 SNPs (Liu et al., unpublished).

Quality control of raw data was performed using Fastq (v. 0.18.0) (15) to obtain high-quality clean reads for subsequent analysis. Then, clean reads from each individual were mapped to the *L. polyactis* genome (GenBank accession: GCA_040670005.1) using BWA (16). GATK (17) was used for genotype detection of target sites to analyze variation; SNPs were filtered with the following settings: deep > 2, miss rate< 0.4, minimum allele frequency > 0.05 using SAMTOOLS (18). ANNOVAR (19) was used for SNP annotation.

### 2.3 Genome-wide association analysis for genetic resistance to VWND

The phenotypic data for GWAS were defined as binary status (BS) (the fish died before half of the total time to death were recorded as 0, and the rest fish were recorded as 1) and survival time (ST) (the time of death). GWAS was performed for identifying SNPs associated with VWND resistance phenotypes using a mixed linear model in GEMMA (http://www.xzlab.org/software.html) software. The Equation 1 used for the mixed linear model is as follows:


(1) Y = Xβ + Zμ + e,

where Y is the vector of the observed phenotypic value; X and Z are the matrixes of the fixed and random additive genetic effects, respectively; β is the coefficient vector; μ is the vector of random animal genetic effects; and e is the vector of the residual effect (20).

The significance threshold was identified via Bonferroni correction. The genome-wide significance association was defined as 0.05 divided by the number of SNPs and suggestive significance association was 1/the number SNPs (21). Specifically, the genome-wide significance association threshold was set to -log_10_(*P*) > 6.29, and the suggestive significance association was set to -log_10_(*P*) > 4.99. Manhattan plots, and quantile-quantile plots were drawn using the qqman package in R (v. 3.6.0). Locations 50 kb upstream and downstream of the above SNPs were searched for candidate genes, which were analyzed in the annotation file of the reference genome. Gene Ontology (GO, http://www.geneontology.org/) functional enrichment analysis was conducted using the online tool DAVID (https://david.ncifcrf.gov/); Kyoto Encyclopedia of Genes and Genomes (KEGG, http://www.genome.jp/kegg/) pathway enrichment analysis was performed with the KOBAS 3.0 online tool (http://kobas.cbi.pku.edu.cn/).

### 2.4 RNA extraction, transcriptome sequencing and analysis

The total RNA of 27 liver tissues from CL, SL, and RL was extracted with Trizol reagent (Invitrogen, Carlsbad, CA, USA) following the manufacturer’s protocol. The quality of RNA was assessed on an Agilent 2100 Bioanalyzer (Agilent Technologies, Palo Alto, CA, USA) and tested via RNase free 1.2% agarose gel electrophoresis. Then, the qualified RNAs were sent to Guangzhou Gene Denovo Biotechnology Co., Ltd. (Guangdong, China) for library construction and sequencing using Illumina Novaseq 6000. To obtain high quality clean reads, raw reads were filtered by the Fastq software. Clean reads were mapped to the reference genome using HISAT 2.2.4 (22) and the mapped reads of each sample were assembled using StringTie (v. 1.3.1) (23) using default settings. For each transcription region, the transcript per kilobase per million mapped reads (TPM) value was calculated using RSEM (24) software to quantify its expression abundance and variations. Principal component analysis (PCA) was performed in R to analyze the relationship among the three groups. Differentially expressed genes (DEGs) were identified by DESeq2 (25) software with the parameters of false discovery rate (FDR)< 0.05 and absolute fold change ≥ 2. To explore the functions of DEGs, GO and KEGG pathway enrichment analyses were performed. All enrichment results use FDR< 0.05 as the threshold for significant enrichment.

### 2.5 Integrated analysis of GWAS and transcriptome data

Candidate genes identified by GWAS were compared with the DEGs in the transcriptome to identify overlaps using Venn diagram. A protein–protein interaction (PPI) network was drawn on the overlapped genes using Cytoscape (v. 3.6.1) based on the interactions identified by the STRING protein interaction database (https://string-db.org/) (26). The R package pheatmap was used to plot a heatmap that describes the changes in expression of target genes after normalization (using z-score) in different samples. A comprehensive analysis was performed combining the overlapping DEGs from different comparison sets (RL vs SL, RL vs CL, or SL vs CL) and the significantly enriched KEGG pathway to identify key genes and important regulatory networks.

### 2.6 Quantitative real-time polymerase chain reaction

To verify the accuracy of GWAS analysis, the five individuals sampled at 96 h after injection with *P. plecoglossicida* were defined as TL96 and the control group sampled simultaneously were defined as CL96. The total RNA of TL96 and CL96 was extracted with Trizol reagent. The cDNA was synthesized by Hifair^®^ III 1st Strand cDNA Synthesis SuperMix (Yeasen, Shanghai, China). A total of 20 candidate genes in GWAS were selected and analyzed by quantitative real-time polymerase chain reaction (qPCR). To investigate the expression levels of cyclic GMP-AMP (cGAMP) synthase (cGAS)-stimulator of interferon genes (STING) signaling pathway and validate the accuracy of the transcriptomic results, the total RNA of CL, SL and RL was extracted and then synthesized. The qPCR was performed on the hub genes of the cGAS-STING pathway (i.e., *cgas*, *sting1*, *tbk1*, *irf3*, *irf7*, *nfκb1*, *p65*, *nfκb2*, *ifnc*, *ifnd*, *ifnh*, *ifnγ*, *il-6*, *il-1β*, *tnfα*), as well as on the seven selected key DEGs. The mRNA expression of these genes was quantified by qPCR using QIAquant 96 2plex (QIAGEN, Germany) and Perfectstart Green qPCR SuperMix (TransGen, Beijing, China). The β-actin gene was used as housekeeping gene, and the primers for target genes were synthesized by Sangon Biotech Co., Ltd. (Shanghai, China; **Supplementary Table S1**). The relative expressions of all detected genes at the mRNA level were calculated following the 2^-ΔΔCt^ method (27).

### 2.7 Western blot analysis

Total proteins of livers were extracted using RIPA lysis buffer (Beyotime, Shanghai, China). These proteins were subjected to a 10% sodium dodecyl sulfatepolyacrylamide gel (SDS-PAGE) and electrophoretically transferred to polyvinylidene fluoride (PVDF) membranes (Solarbio, Beijing, China). Subsequently, the membranes were blocked with 5% skim milk for 1 hour at room temperature, prior to overnight incubation at 4 °C with a *Lp*STING1 rabbit polyclonal antibody (dilution 1:1000; generated in-house) and an Actin rabbit antibody (dilution 1:3000; ABconal, Wuhan, China). Following three washes of 15 minutes each with Tris-buffered saline containing Tween (TBST) (composition: 20 mM Tris, 150 mM NaCl, 0.05% Tween-20), the membranes were incubated with horseradish peroxidase (HRP)-linked goat anti-rabbit (1:3000; Beyotime, Shanghai, China) for 1 hour at 37 °C. The signals detected by chemiluminescence using an enhanced chemiluminescence (ECL) reagent (Millipore, MA, USA), and captured with the Tanon 5200 imaging system (Tanon, Shanghai, China). The ImageJ software (v. 1.8.0) was used to quantify the densitometry of the protein bands on the immunoblots. Data were normalized to the level of the Actin protein. This experimental procedure was replicated three times.

### 2.8 Statistical analysis

All results were expressed as mean ± standard error of the mean (mean ± SEM). Statistical differences were determined with one-way ANOVA and Tukey’s multiple range tests using SPSS 22.0 software. *P*< 0.05 indicated that the difference was significant, and *P*< 0.01 indicated that the difference was extremely significant.

## 3 Results

### 3.1 Phenotype statistics and bacterial challenge

**Figure 1:**
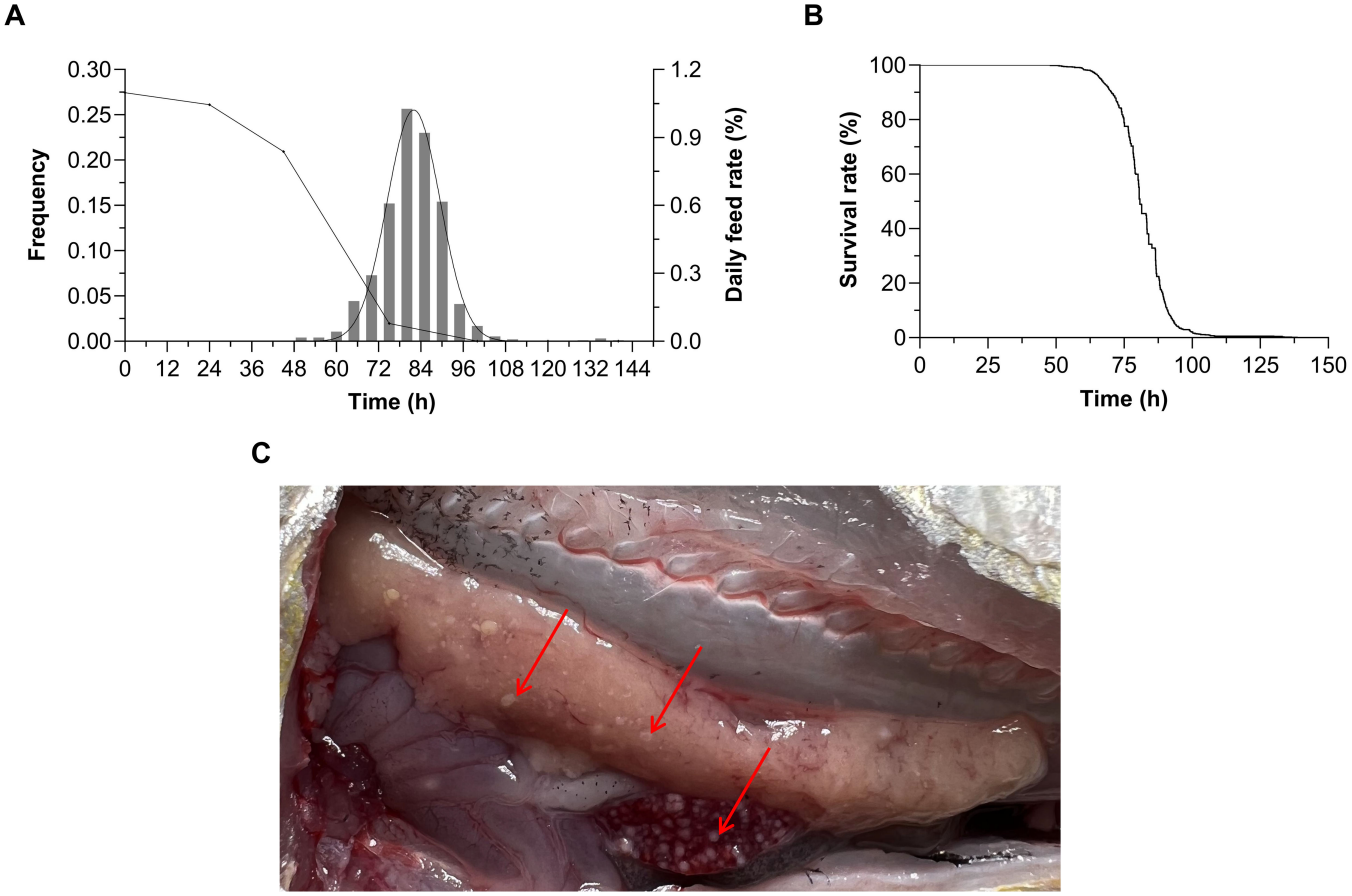
**(A)** Frequency distribution and daily feeding rate change curves under *Pseudomonas plecoglossicida* infection in *Larimichthys polyactis.* **(B)** Survival curve of 946 *L. polyactis* after *P. plecoglossicida* infection. The evolution in survival rate showed a trend of firstly slowly decreasing, then rapidly declining, and finally slowly decreasing to zero. **(C)** The dissection symptoms of visceral white-nodules disease in *L. polyactis.* Red arrows indicate white bacterial nodules in the liver and spleen.

After excluding 4 individuals that died due to non-experimental treatments (defined as death within 30 minutes post-injection accompanied by opercula flaring and body rigidity), the effective sample of *P. plecoglossicida* challenge was 946 fish. The results showed that the frequency distribution of survival time was nearly normally distributed (**Figure 1A**). As shown in **Figure 1B**, the evolution in survival rate showed a trend of firstly slowly decreasing, then rapidly declining, and finally slowly decreasing to zero. The challenge test lasted for 138.5 h and experimental fish began to die 47.98 h after infection. The average survival time was 81.48 h and deaths peaked at 75–90 h. During the challenge experiment, the daily feeding rate decreased rapidly on the third day after infection, after which fish began to stop feeding entirely. At autopsy, symptoms of VWND were found to be abundant in the late stage of infection (**Figure 1C**). These observations are similar to the incidence condition in farming.

### 3.2 Genotyping and population structure analyses

Injected *L. polyactis* were genotyped using 100 K liquid chips. After filtration and quality control, 98,603 high-quality SNPs were obtained. The locus detection rate of samples ranged from 95.15% to 98.87%, with an average of 98.08% (**Supplementary Figure S2**). The genotypic concordance rate of all three duplicated samples exceeded 98.00%, which ensured the accuracy of the genotype. As shown in **Supplementary Figure S3**, the fish died before half of the total time to death were recorded as 0 (red triangles), and the rest fish were recorded as 1 (blue triangles). The results of PCA based on high-quality SNPs indicated that the experimental population was evenly distributed without stratification.

### 3.3 GWAS to identified the candidate genes

**Figure 2:**
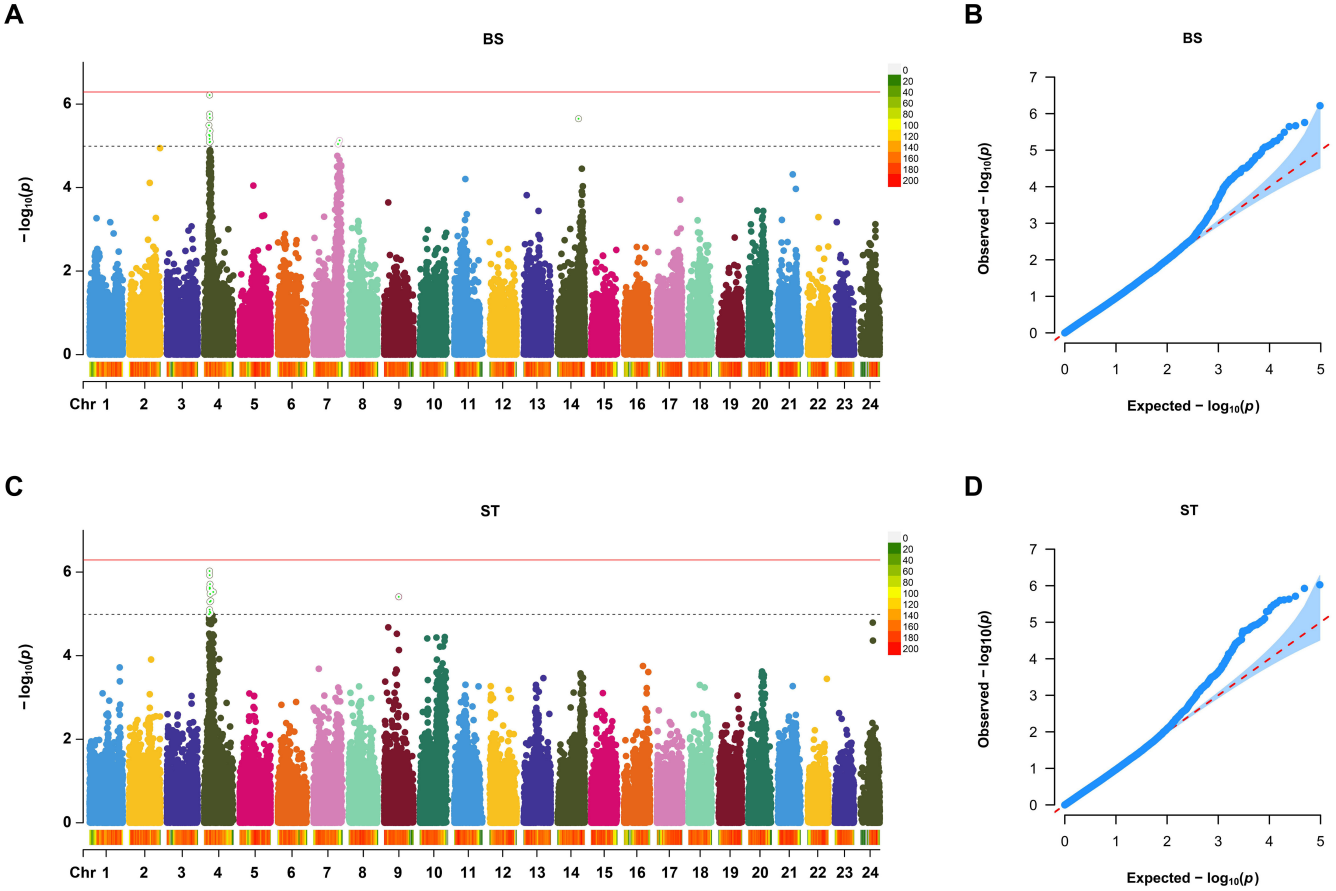
Manhattan plot and Quantile-Quantile (Q-Q) plot of genome-wide association study (GWAS) for resistance to VWND in *L. polyactis*. Manhattan plot of GWAS for **(A)** binary status (BS) and **(C)** survival time (ST). The X-axis represents the chromosomes, and the Y-axis shows the –log_10_(*p*). The red solid line represents genome-wide significance association threshold. The dashed line represents the suggestive significance association. Q-Q plot of GWAS for **(B)** BS and **(D)** ST. The X-axis represents the expected –log_10_(*p*), and the Y-axis represents the observed –log_10_(*p*). A total of 13 SNPs of suggestive significance association level were distributed on Chromosome 4, 7, and 14 in the BS trait. A total of 15 suggestive significantly associated SNPs were located on Chromosomes 4 and 9 in the ST trait.

Based on the suggestive association threshold, a total of 22 SNPs were found to be associated with VWND resistance (**Table 1**). Of these, 13 SNPs of suggestive significance association level were distributed on Chromosome 4, 7, and 14 in the BS trait (**Figure 2A**). Furthermore, 15 suggestive significantly associated SNPs were located on Chromosomes 4 and 9 in the ST trait, six of which were repeated in the BS trait (**Figure 2C**). The SNPs with the strongest association with disease resistance were Chr4-5527145 and -5488677 in the BS and ST traits, respectively. Quantile-quantile plots showed that the statistical model was reasonable and the results were reliable (**Figure 2B, D**).

**Table 1:** Information of suggestively significant single-nucleotide polymorphisms for visceral white-nodules disease resistance. .

Trait	Chr	Position	Ref	Alt	Maf	-log_10_ (P-value)	Location
BS	4	4822370	T	C	0.45	5.49	intronic
	4	4924712	T	C	0.50	5.26	downstream
	4	5482236	T	C	0.48	5.08	intronic
	4	5498543	C	A	0.48	5.16	intronic
	4	5527145	C	G	0.50	6.22	intronic
	4	5580670	G	A	0.38	5.76	intronic
	4	5590046	T	C	0.38	5.24	intronic
	4	5748997	A	G	0.50	5.67	intronic
	4	5922208	T	C	0.38	5.35	downstream
	4	6055098	C	T	0.39	5.10	intronic
	7	24996538	T	C	0.31	5.04	intergenic
	7	26620846	T	A	0.31	5.13	intergenic
	14	21715033	C	A	0.14	5.65	intergenic
ST	4	4962096	T	A	0.38	4.99	intergenic
	4	5488677	A	C	0.20	6.03	intronic
	4	5511267	T	C	0.39	5.10	intronic
	4	5527145	C	G	0.50	5.60	intronic
	4	5580670	G	A	0.38	5.93	intronic
	4	5590046	T	C	0.38	5.63	intronic
	4	5748997	A	G	0.50	5.29	intronic
	4	5840313	C	G	0.28	5.05	intronic
	4	5922208	T	C	0.38	5.61	downstream
	4	5929171	A	G	0.38	5.71	downstream
	4	6055098	C	T	0.39	5.48	intronic
	4	6332463	C	T	0.42	5.03	downstream
	4	6661160	C	T	0.41	5.31	intronic
	4	9223910	G	A	0.28	5.53	downstream
	9	14705694	A	T	0.00	5.41	intergenic

The underline position means this single-nucleotide polymorphism was also co-identified in both traits.

Candidate genes associated with VWND resistance were searched around a ±50 kb region of the above SNPs in the reference genome, and a total of 60 potential candidate genes were identified (**Supplementary Table S2**). For the BS trait, the most enriched GO terms included chloride transport, inorganic anion transport, ribosomal small subunit binding, and Hrd1p ubiquitin ligase complex (**Supplementary Figure S4A**). KEGG enrichment analysis showed that glycosphingolipid biosynthesis - ganglio series, fatty acid biosynthesis, p53 signaling pathway, apoptosis, and ubiquitin mediated proteolysis were enriched in the BS trait (**Supplementary Figure S4B**). For the ST trait, GO enrichment analysis showed that genes are mainly involved in gamma-aminobutyric acid signaling pathway, activation of innate immune response, GABA receptor activity, and ER ubiquitin ligase complex (**Supplementary Figure S4C**). KEGG enrichment analysis showed that ubiquitin mediated proteolysis, protein processing in endoplasmic reticulum, FoxO signaling pathway, cytosolic DNA-sensing pathway, and MAPK signaling pathway were enriched in the ST trait (**Supplementary Figure S4D**).

Interestingly, for both disease resistance traits, most of the suggestive significantly associated SNPs were concentrated on Chromosome 4, commonly located at the loci of Chr4-5527145, -5580670, -5590046, -5748997, -5922208, and -6055098. These regions included 24 potential candidate gene, such as histone deacetylase 3 (*hdac3*), G protein-coupled receptor 137 (*gpr137*), phospholipase D family member 3 (*pld3*), family with sequence similarity 199, X-linked (*fam199x*), LON peptidase N-terminal domain and ring finger protein 3 (*lonrf3*), and cathepsin F (*ctsf*).

### 3.4 Transcriptome analysis to uncover VWND-resistance related genes

**Figure 3:**
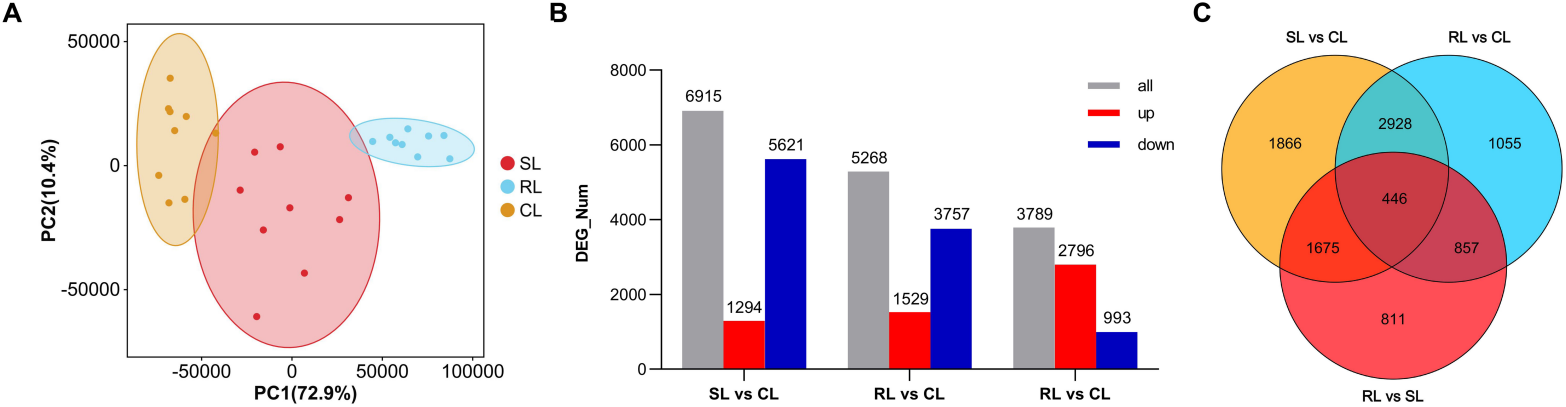
**(A)** Principal component analysis of the control group (CL), sensitive group (SL), and resistant group (RL). Orange, red, and blue points represent each fish of the CL, SL, and RL, respectively. Ellipses represnt 95% confidence ellipses. PC1: first principal components; PC2: second principal components. **(B)** Number distribution and expression trend of differentially expressed genes (DEGs) among CL, SL, and RL. Red bar: Significantly upregulated DEGs; Blue bar: Significantly downregulated DEGs. **(C)** Venn diagram showing the overlap of DEGs among SL vs CL, RL vs CL and RL vs SL.

As shown in **Supplementary Figure S5A**, the sensitive fish (SL) were sampled from 43.38 to 51.83 h post infection and the resistant fish (RL) were sampled form 131.42 to 165.42 h post infection. The mean survival times in SL and RL groups were 47.06 ± 3.10 h and 138.90 ± 9.95 h, respectively (**Supplementary Figure S5B**). Using the Illumina paired-end RNA-seq approach, transcriptome sequencing generated 1,295,621,636 raw reads. The raw RNA-seq data were deposited in the NCBI sequence read archive database under accession number PRJNA1142980. For the CL, SL, and RL groups, filtering low-quality reads yielded 440,624,088, 401,078,390, and 443,937,350 clean reads, with Q20 scores of 97.89, 97.98, and 97.84, respectively (**Supplementary Tables S3, S4**). The total mapped reads ratios were 90.17%, 87.33%, and 89.02% in CL, SL and RL groups, respectively. There was a significantly difference between both groups (*P*< 0.01). The results of PCA disclosed a certain degree of separation among CL, SL, and RL groups, indicating that the differences among groups increased after infection; also, SL and RL samples might have different transcriptional patterns against VWND (**Figure 3A**).

Compared to the CL group, 6,915 and 5,286 DEGs were obtained in the SL and RL groups, respectively. Among these DEGs, 1,294 upregulated and 5,621 downregulated genes were identified between SL and CL groups, respectively, and 1,529 upregulated and 3,757 downregulated genes were identified between RL and CL groups, respectively. In addition, 3,789 DEGs were identified between the RL and SL groups, including 2,796 upregulated and 993 downregulated genes (**Figure 3B**). Analysis of the intersection of different DEG sets by a Venn diagram showed that 1,675 DEGs only overlapped between SL vs CL and RL vs SL. This mode of overlap suggests that these DEGs were only significantly differentially expressed in the SL group in response to *P. plecoglossicida* infection; 857 DEGs only overlapped between RL vs CL and RL vs SL, suggesting that these DEGs were only significantly differentially expressed in the RL group in response to *P. plecoglossicida* infection. Moreover, 446 DEGs overlapped among SL vs CL, RL vs CL, and RL vs SL, suggesting that both in the SL and RL group, these DEGs were significantly differentially expressed in response to *P. plecoglossicida* infection (**Figure 3C**).

To identify the functions of the above three types of DEG sets, functional enrichment analysis was performed. The top GO enrichment results showed that 1,675 DEGs overlapped between SL vs CL and RL vs SL were significantly enriched in cytoplasm, endosome, intracellular anatomical structure, cytoplasmic vesicle, intracellular vesicle, and lysosome (**Supplementary Figure S6A**). However, most of the 857 DEGs overlapped between RL vs CL and RL vs SL were significantly enriched in immune-related terms, such as response to bacteria, immune response, response to biotic stimuli, defense response, cytokine-mediated signaling pathway, and immune system process (**Supplementary Figure S6B**). These results indicate that the RL group may have a stronger and longer lasting immune response. Interestingly, the co-overlapping 446 DEGs among SL vs CL, RL vs CL, and RL vs SL were significantly enriched in metabolic process terms, including organic acid metabolic process, oxoacid metabolic process, carboxylic acid metabolic process, small molecule metabolic process, and fatty acid metabolic process (**Supplementary Figure S6C**).

**Figure 4:**
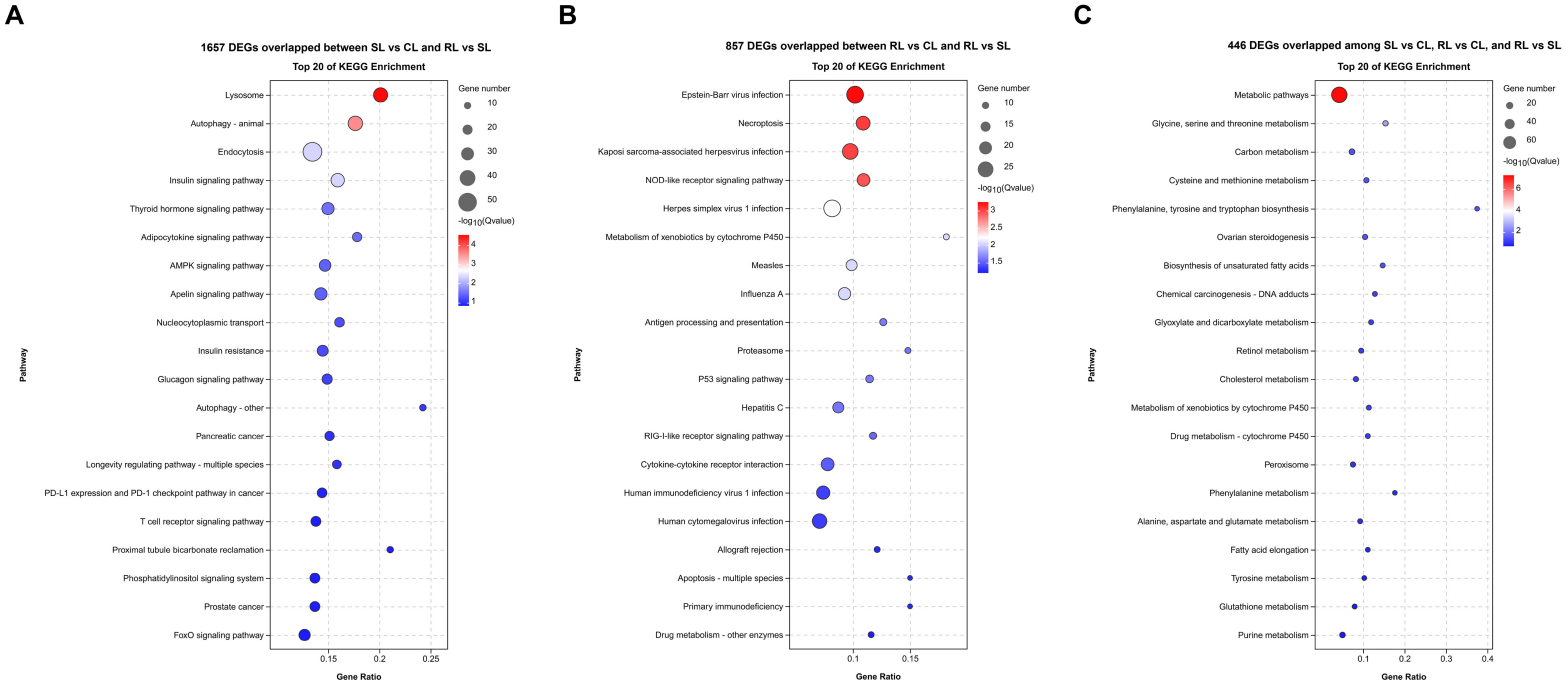
Difference of transcriptomic expression patterns among CL, SL, and RL after infection. **(A–C)** Bubble plot of the top 20 significantly enriched KEGG pathways of 1657 DEGs overlapped between SL vs CL and RL vs SL, 857 DEGs overlapped between RL vs CL and RL vs SL and 466 DEGs overlapped among SL vs CL, RL vs CL, and RL vs SL, respectively.

KEGG enrichment analysis showed that most of the 1,675 DEGs overlapped between SL vs CL and RL vs SL were significantly enriched in lysosome, autophagy - animal, endocytosis, insulin signaling pathway, thyroid hormone signaling pathway, adipocytokine signaling pathway, and AMPK signaling pathway (**Figure 4A**). The 857 DEGs overlapped between RL vs CL and RL vs SL were significantly enriched in Epstein-Barr virus infection, necroptosis, nucleotide-binding oligomerization domain (NOD)-like receptor signaling pathway, p53 signaling pathway, cytokine-cytokine receptor interaction, and apoptosis (**Figure 4B**). Moreover, many of the 446 DEGs overlapped among SL vs CL, RL vs CL, and RL vs SL were significantly enriched in metabolic-related pathways, including metabolic pathways, glycine, serine, and threonine metabolism, carbon metabolism, cysteine and methionine metabolism, phenylalanine, tyrosine and tryptophan biosynthesis, ovarian steroidogenesis, and biosynthesis of unsaturated fatty acids. (**Figure 4C**).

### 3.5 GWAS and transcriptome were combined to screen the key candidate genes related to VWND resistance

**Figure 5:**
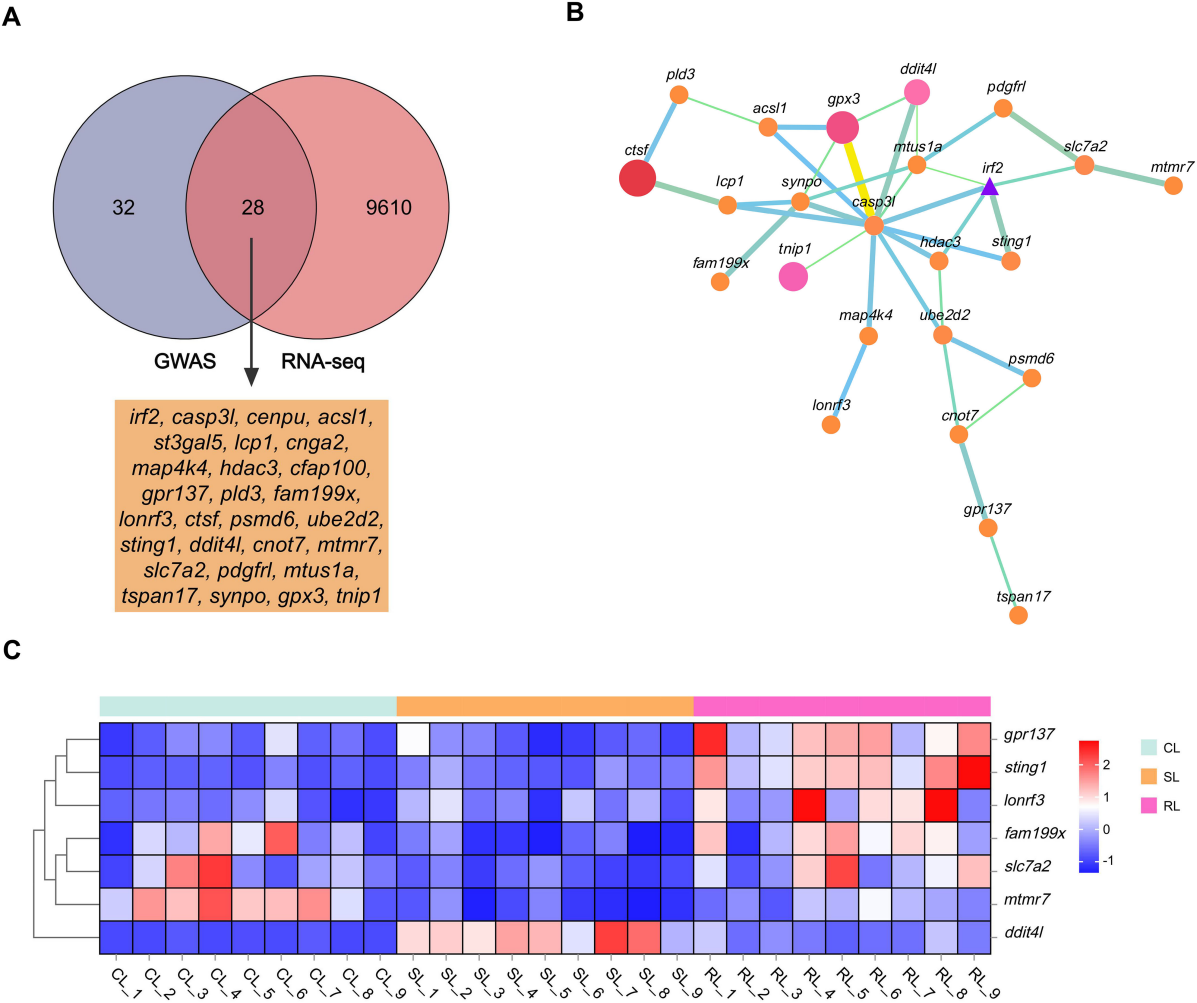
**(A)** Venn diagram between the 9,638 DEGs of RNA-seq and the 60 potential candidate genes of GWAS. The overlap genes were identified as the final candidate genes. **(B)** Protein–protein interaction network of candidate genes. Node circles size and colour are positively correlated with abundance of interacting genes. Line segments thickness and colour are positively correlated with protein interactions. Triangle means transcription factor. **(C)** Heat map analysis of the seven key genes in livers of CL, SL, and RL, respectively. Genes in red are upregulated while those in blue are downregulated.

To identify candidate genes and potential mechanisms associated with VWND resistance in *L. polyactis*, a cross-analysis of the gene expression results and genome-wide suggestive significant SNPs was conducted. By comparing 60 potential candidate genes in GWAS to the all 9,638 DEGs in the transcriptome, 28 DEGs were identified as the final candidate genes (**Figure 5A** **,** **Table 2**). All of these were predicted by suggestive significant SNPs on Chromosome 4, focusing on Chr4-4924712, -4822370, -5488677, -5527145, -5580670, -6332463, -6661160, and -9223910. Interestingly, these included the SNPs most significantly associated with VWND resistance in the BS and ST traits. A PPI analysis on the 28 candidate genes was conducted and a PPI network was constructed (**Figure 5B**). Its core contained 24 protein interactions, and hub genes included *casp3l*, *synpo*, *gpx3*, *mtus1a*, *irf2*, *sting1*, and *hdac3*. Based on the overlap of DEGs (including RL vs SL, SL vs CL, or RL vs CL), seven candidate genes (i.e., *ddit4l, fam199x, mtmr7, slc7a2, gpr137, lonrf3*, and *sting1*) were identified as key genes associated with resistance to VWND. Heat map analysis showed that *gpr137*, *lonrf3*, *fam199x*, and *sting1* were more highly expressed in the RL group compared to the other genes (**Figure 5C**). It is worth noting that *sting1* is involved in the NOD-like receptor signaling pathway where the 857 DEGs were significantly enriched.

**Table 2:** The 28 candidate genes associated with visceral white-nodules disease resistance.

SNP	Gene	Overlap of DEGs	Function
Chr4-4822370	*irf2*	SL vs CL	Transcription regulatory region sequence-specific DNA binding
	*acsl1*	SL vs CL	Lipid transport and metabolism
	*cenpu*	SL vs CL, RL vs CL	Lipid transport and metabolism
	*st3gal5*	SL vs CL, RL vs CL	Glycosphingolipid biosynthesis
	*casp3l*	SL vs CL, RL vs CL	Apoptosis
Chr4-4924712	*lcp1*	RL vs CL	Calcium ion binding
	*cnga2*	RL vs CL	Ion channel activity
Chr4-5482236, -5488677, -5498543 -5511267, -5527145	*hdac3*	RL vs SL	Metal ion binding
	*map4k4*	SL vs CL, RL vs CL	Signal transduction mechanisms
	*cfap100*	SL vs CL, RL vs CL	Motile cilium
Chr4-5482236, -5488677, -5498543, -5511267, -5527145, -5580670	*gpr137* ^#^	RL vs CL, RL vs SL	Regulation of signal transduction
Chr4-5580670, -5590046	*lonrf3* ^#^	RL vs CL, RL vs SL	Protein ubiquitination
Chr4-5488677, -5527145, -5580670, -5590046	*fam199x* ^#^	SL vs CL, RL vs SL	Cell adhesion and migration
Chr4-5488677, -5498543, -5511267, -5527145, -5580670, -5590046	*pld3*	RL vs SL	Lipid transport and metabolism
Chr4-5922208, -5929171	*ctsf*	SL vs CL, RL vs CL	Apoptosis
Chr4-6055098	*psmd6*	SL vs CL	Posttranslational modification, protein turnover, chaperones
Chr4-6332463	*ube2d2*	RL vs SL	Ubiquitin mediated proteolysis
	*ddit4l* ^#^	SL vs CL, RL vs CL, RL vs SL	Regulation of signal transduction
	*sting1* ^#^	RL vs CL, RL vs SL	Innate immune response
Chr4-6661160	*mtmr7* ^#^	SL vs CL, RL vs SL	Inositol phosphate metabolism
	*slc7a2* ^#^	SL vs CL, RL vs SL	Amino acid transport and metabolism
	*cnot7*	SL vs CL, RL vs CL	RNA degradation
	*pdgfrl*	SL vs CL, RL vs CL	Signal transduction mechanisms
	*mtus1a*	SL vs CL, RL vs CL	Cell proliferation
Chr4-9223910	*tspan17*	SL vs CL	Ubiquitin-protein transfer
	*gpx3*	RL vs CL	Response to oxidative stress
	*tnip1*	RL vs CL	Autoimmunity and tissue homeostasis
	*synpo*	SL vs CL, RL vs CL	Tight junction

^#^ means this gene was identified as a key gene.

### 3.6 qPCR verification results

**Figure 6:**
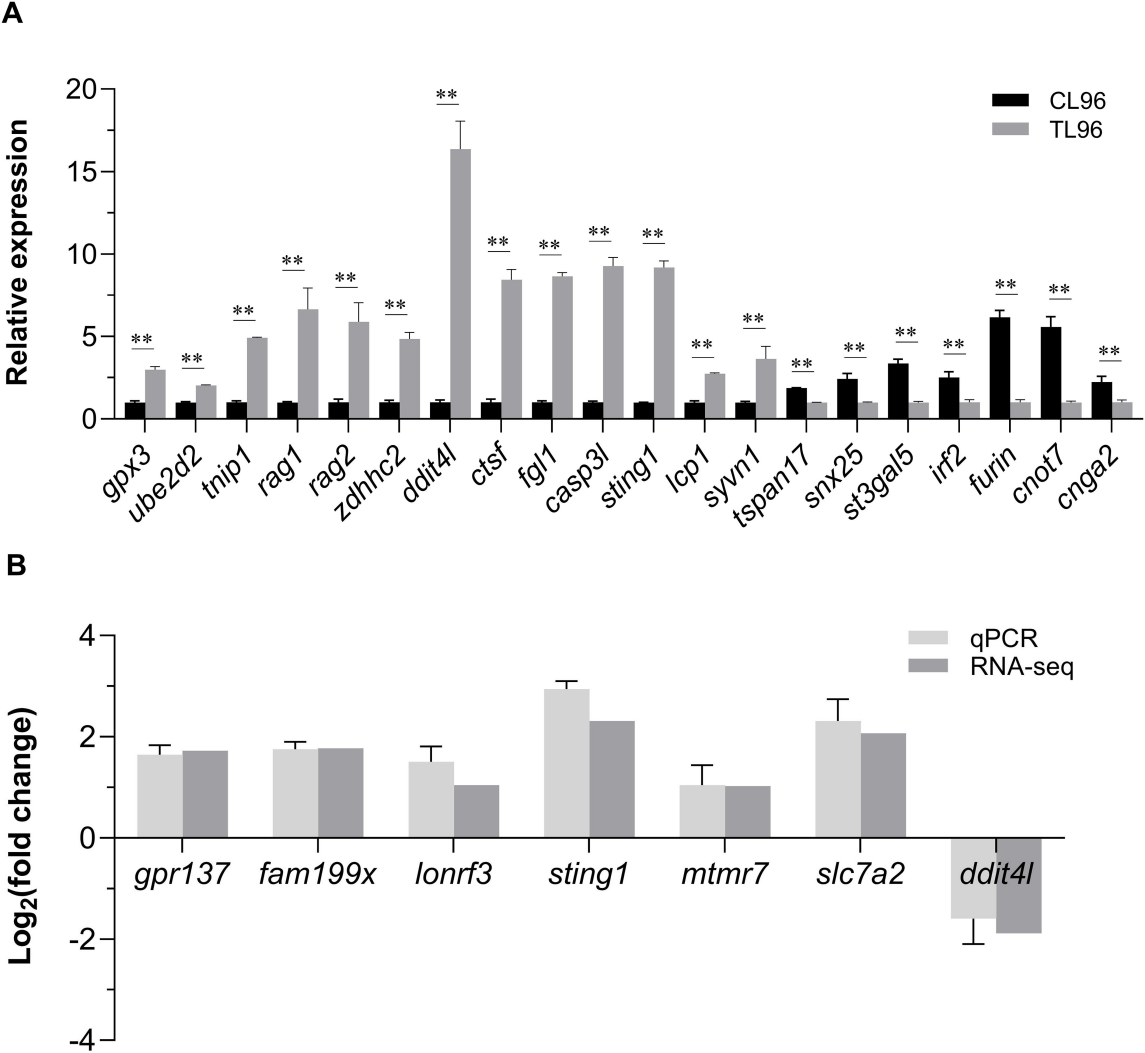
Quantitative real-time polymerase chain reaction validation of the GWAS and RNA-Seq results. **(A)** Expression of 20 potential candidate genes from GWAS at 96 h after infection in *L. polyactis*. ** means an extremely significant difference (*P*< 0.01) compared with the control group. **(B)** Relative expression values from quantitative real-time polymerase chain reaction results and transcripts per million values in the RNA-seq were compared. X-axis represents the seven key genes name, and Y-axis represents the expression levels (Log_2_(fold change)) in RL relative to SL. Data were presented as means ± SEM.

Twenty candidate genes were selected to confirm the GWAS results by qPCR. The results showed that at 96 h after infection, 13 genes exhibited significantly higher expressions and the other seven genes exhibited significantly lower expressions in the liver compared to the control group (*P*< 0.01) (**Figure 6A**). This result indicates that the GWAS data have high reliability. Furthermore, the qPCR results of the seven key genes were highly consistent with the gene expression data from the transcriptome (**Figure 6B**). The Pearson’s correlation coefficient of qPCR and RNA-seq was 0.97 (*P*< 0.01), indicating the high accuracy of the RNA-seq data.

### 3.7 Analysis of key gene and protein expression in the cGAS-STING signaling pathway

**Figure 7:**
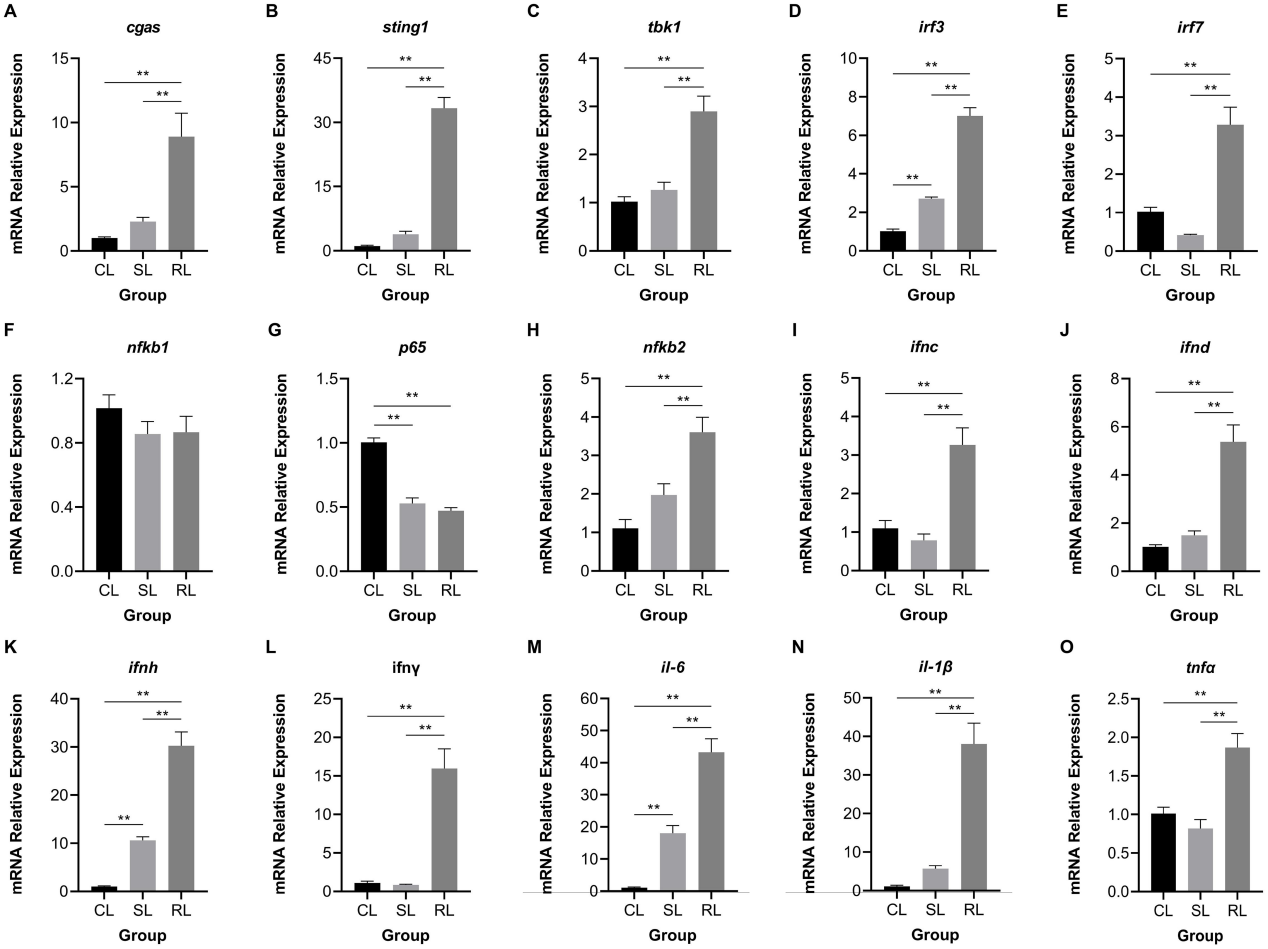
Comparison of expression patterns of hub genes in the cGAS-STING signaling pathway among CL, SL, and RL following infection. **(A)** *cgas*, **(B)** *sting1*, **(C)** *tbk1*, **(D)** *irf3*, **(E)** *irf7*, **(F)** *nfκb1*, **(G)** *p65*, **(H)** *nfκb2*, **(I)** *ifnc*, **(J)** *ifnd*, **(K)** *ifnh*, **(L)** *ifnγ*, **(M)** *il-6*, **(N)** *il-1β*, and **(O)** *tnfα*. The expression levels of *cgas*, *sting1*, *tbk1*, *irf3*, *irf7*, *nfκb2*, *ifnc*, *ifnd*, *ifnh*, *ifnγ*, *il-6*, *il-1β*, and *tnfα* were significantly upregulated in the RL group; *nfκb1* mRNA expression levels showed no significant difference among the three groups; *p65* mRNA expression levels was significantly downregulated in the RL group. Data were presented as means ± SEM. ** means an extremely significant difference (*P*< 0.01) compared with the control group or the SL group.

**Figure 8:**
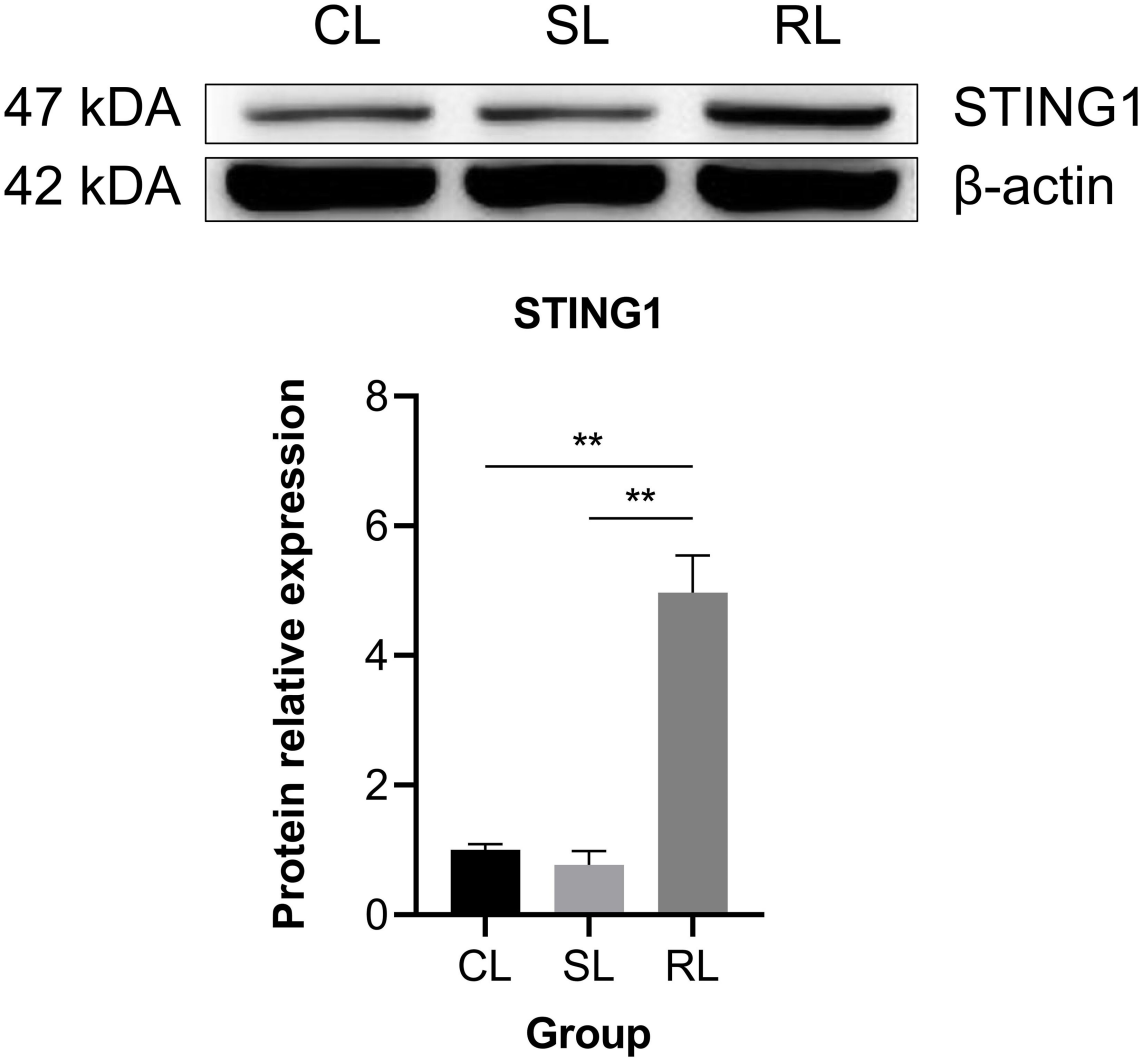
Comparison of protein expression level of STING1 among CL, SL, and RL following infection. Data were presented as means ± SEM. ** means an extremely significant difference (*P*< 0.01) compared with the control group or the SL group. The protein expression levels of STING1 in the RL group were significantly higher than those in the CL and SL groups.

Fifteen hub genes of cGAS-STING signaling pathway were selected to investigate the differential expression patterns among CL, SL, and RL following infection. The results showed that RL exhibited a stronger response in the cGAS-STING pathway compared to CL and SL. Specifically, the mRNA expression levels of *cgas*, *sting1*, *tbk1*, *irf3*, *irf7*, *nfκb2*, *ifnc*, *ifnd*, *ifnh*, *ifnγ*, *il-6*, *il-1β*, and *tnfα* were significantly upregulated in the RL group (*P*< 0.01) (**Figure 7**). In contrast, *nfκb1* mRNA expression levels showed no significant difference among the three groups, while *p65* mRNA expression levels was significantly downregulated in the RL group. The results of the Western blot analysis revealed that the protein expression levels of STING1 in the RL group were significantly higher than those in the CL and SL groups (*P*< 0.01) (**Figure 8**).

## 4 Discussion

### 4.1 Genetic architecture of VWND resistance

Due to low cost in sequencing, GWAS has been widely used to discover variants and functional genes as well as to understand genomic mechanisms underpinning disease resistance in aquatic animals. In *O. mykiss*, a significant quantitative trait locus was detected on Chromosome 3 for *Flavobacterium columnare* resistance, along with several suggestive quantitative trait loci on two other chromosomes (28). Thirty-three SNPs significantly associated with *Vibrio harveyi* resistance were detected in *Cynoglossus semilaevis* and several genes (i.e., *plekha7*, *nucb2*, and *fgfr2*) were also found to potentially play roles in disease resistance (29). In this study, 22 SNPs and 60 candidate genes were identified by GWAS, which were distributed on Chromosomes 4, 7, and 9. The resistance to VWND in this population was confirmed to have a polygenic architecture, as is common for disease resistance traits (10, 30). In *L. crocea*, a total of 5 resistance-associated SNPs and 13 candidate genes were identified and located on chromosomes LG3, LG11, LG15, and LG21, respectively. And the results pointed toward a polygenic architecture of resistance to VWND, but potentially include some candidate regions which explain moderate levels of the genetic variation (14). This different mainly due to the species difference between *L. crocea* and *L. polyactis*. Moreover, an analysis using BS and ST traits identified six identical SNPs. This result indicates that there is a degree of similarity between both results. Similar results were also obtained in the GWAS analysis of resistance against *Piscirickettsia salmonis* in *S. salar* using binary survival and time to death traits (31). Barría et al. (32) also identified the same SNPs (AX317616757) with the strongest association with disease resistance for BS and time to death, which is located in the second intron of the *lgals17* gene.

Although most immune-related genes had elevated levels of expression after infection, downregulation of certain immune-related genes (i.e. *tspan17*, *snx25*, *st3gal5*, *irf2*, *furin*, *cnot7*, and *cnga2*) was found in VWND infected fish compared to CL96. Similar patterns have also been reported in *Pelteobagrus vachelli* infected with *Edwardsiella ictaluri* (33) and *S. salar* suffered *Neoparamoeba perurans* infection (34). Gervais et al. (11) revealed that the complement and coagulation pathway and several genes (i.e. *irf1*, *irf4* or *irf8*) in the interferon pathway was down-regulated in which Infectious Salmonid Anaemia Virus infected *S. salar*. Besides, Boison et al. (13) found the genes encoding chemokine ligands such as *ccl4*, *ccl19*, *cxcl9*, and *cxcl10*, were significantly down-regulated in amoebic gill disease infected fish compared to the naïve *S. salar*. Such a downregulation pattern of immune response and immunosuppression of the immune system of the host might be a means through which *P. plecoglossicida* restricts host immune regulation and facilitates *L. polyactis* infection. The ubiquitin mediated proteolysis pathway was co-enriched in both traits; this pathway controls a wide range of essential cellular processes including cell cycle, cell death, inflammatory signaling, and defense against pathogens (35). Balasubramaniam et al. (36) found that ubiquitin mediated proteolysis supports the innate immune mechanism to overcome bacterial infection. Thus, ubiquitin mediated proteolysis may be essential to fight off bacterial pathogens in *L. polyactis.*

### 4.2 Transcriptomic signatures of VWND resistance

Comparative transcriptional analysis using a Venn diagram identified three intersections of different DEGs sets. A total of 1,675 DEGs were differentially expressed in both SL vs CL and RL vs SL, but these were non-differentially expressed in RL vs CL. This result suggests that these genes could be related to susceptibility to VWND. Lysosomes are key organelles for cell homeostasis, metabolic control, and environmental adaptation, facilitating degradation of both intracellular (via autophagy) and extracellular (via endocytosis) materials (37). The 1,675 DEGs were significantly enriched in lysosome, autophagy, and endocytosis in this study, suggesting that susceptibility to VWND could be connected with imbalance in cellular homeostasis. Additionally, autophagy plays an important role in inflammation through effecting the survival, development, and homeostasis of inflammatory cells (e.g., macrophages, neutrophils, and lymphocytes) (38). Bai et al. (39) found that autophagy may play a key role in the regulation of the formation of chronic inflammatory granulomas. Likewise, white nodules caused by *P. plecoglossicida* in the present study are also considered as granulomas. Therefore, it can be speculated that the deregulation of autophagy can contribute to the susceptibility of *L. polyactis.*

A total of 857 DEGs were differentially expressed in both RL vs CL and RL vs SL, but these were non-differentially expressed in SL vs CL, suggesting an association between these genes and resistance to VWND. These DEGs were significantly enriched in p53 signaling pathway, cytokine-cytokine receptor interaction and apoptosis, which are closely linked and interact with each other. Cytokines play an important role in the immune response during bacterial infections (40). Endotoxins of Gram-negative bacteria (lipopolysaccharide, LPS) are particularly inflammatory because they activate related cytokines and inflammasomes (41). Baoprasertkul et al. (42) revealed differences in *cxc* chemokine expression profiles between resistant and susceptible *Ictalurus punctatus* after infection of *E. ictaluri*. Moreover, pro-inflammatory cytokines can activate immune pathways such as nuclear factor kappa B (NF-κB) and play a certain role in the antibacterial responses of host (43). In this study, *il-6*, *il-6st*, *il-17rc*, *ifn-ar2*, *tnf*, and *cxcr3* were significantly upregulated in the RL group, which suggests that *P. plecoglossicida* induces an inflammatory response to activate immune cascades

Apoptosis is the cumulative response of p53 signaling pathway and numerous signals, such as *caspase-7* and *caspase-8*, to bacterial infection-induced cellular stress and destruction of inflammatory tissue (44, 45), which is crucial to eliminate redundant or damaged cells, as well as to maintain homeostasis in the body (46). However, dysregulation of apoptosis, inappropriate activation, or inhibition can lead to many pathological conditions and diseases (47). For example, the p53 signaling pathway was significantly enriched in the susceptible control populations of *L. crocea* which has a lower survival rate, and may resulted in an apoptotic response because of irreversible cell damage caused by *P. plecoglossicida* infection (14). Cell death depends on the balance between the activities of pro- and anti-apoptotic factors. In the present study, *caspase-7*/*8, birc2*/*5*, and *xiap* were highly expressed in the RL group. Interestingly, the inhibitor of apoptosis BIRC2 may induce signaling to the inflammatory transcription factor NF-κB and protection from cell death (48). XIAP, a member of the inhibitor of apoptosis protein family, not only suppresses caspases, but also regulates inflammatory signaling (49). As an immune-related gene, *birc5* negatively regulates apoptosis or programmed cell death by inhibiting caspase activation and promoting cell proliferation (50). This evidence suggests the existence of a balance mechanism between inflammatory response and apoptosis that maintains host survival following bacterial invasion.

A total of 446 DEGs were differentially expressed in SL vs CL, RL vs CL, and RL vs SL, and were significantly enriched in pathways related to metabolism. Similar to certain previous studies, in diseased fish, metabolic processes were strongly affected by *P. plecoglossicida* infection (51). Several energy metabolism pathways are downregulated in response to infection. This may be a result of diversion of cellular resources towards a physiological response of the host to adjust cellular homeostasis or to reduce appetite. This has also been suggested in previous research on the response of macrophage cell lines to *P. salmonis* infection (52). Nonetheless, pathogens also reprogram the cellular metabolism of infected cells, further causing metabolic dysregulation (53, 54). These are excellent examples of host-pathogen interactions. Notably, significantly enriched pathways are almost always amino acid metabolic pathways, including glycine, serine, and threonine metabolism, cysteine and methionine metabolism, phenylalanine, tyrosine, and tryptophan biosynthesis, phenylalanine metabolism, alanine, aspartate, and glutamate metabolism and tyrosine metabolism. As it is reported that the amino acids and their metabolites modulate the innate immune response of aquatic animals (55, 56). A dependency of *P. salmonis* on amino acid metabolism in *S. salar* was reported, implying novel mechanisms of pathogenesis based on the capacity to uptake nutrients from the host (57). Therefore, amino acid metabolism had different response features between pre- and post-infection and between susceptible and resistant groups. This difference indicates that the strategies of amino acid metabolism in the host are closely related to resistance to bacterial infections.

### 4.3 Key regulatory mechanisms associated with VWND resistance

Transcriptome sequencing showed that different gene expression patterns exist between RL and SL groups, which partly validate the accuracy of the GWAS results (58). By combining GWAS and RNA-seq, 28 DEGs were identified as the final candidate genes. Then based the DEGs of RL vs SL and considering the overlap of DEGs from different sets, seven key genes (i.e., *ddit4l, fam199x, mtmr7, slc7a2, gpr137, lonrf3*, and *sting1)* highly related to disease resistance were further identified. Specifically, *gpr137*, *lonrf3*, and *sting1* were identified in the overlap of RL vs SL and RL vs CL, *fam199x*, *mtmr7* and *slc7a2* were identified in the overlap of RL vs SL and SL vs CL, *ddit4l* were identified in the overlap of RL vs SL, RL vs CL and SL vs CL. Their functions include signal transduction, cell adhesion, metabolism, ubiquitination, and immune response. Moreover, *fam199x, lonrf3*, and *gpr137* are co-located in BS and ST traits. *Fam199x* is known to be strongly associated with human disease (59), but its function and mechanism in disease remain unclear. Evidence also indicates that under heat stress, novel-lncRNAs of chicken were significantly co-expressed with *fam199x* and *lonrf3* (60). *Lonrf3* has been associated with Alzheimer’s disease (61) and pancreatic cancer (62). However, the specific mechanism of *lonrf3* has not been reported and only one related study indicated that it could be related to the dysregulation of the ecology of gut microbiota (63). The orphan receptor GPR137 is an integral membrane protein involved in several types of cancer (64). For example, *gpr137* plays pro-oncogenic roles in ovarian cancer through regulating the PI3K/AKT pathway (65). Besides, Gan et al. (66) found *gpr137*-knockout cells exhibited defective autophagy and an expanded lysosome compartment, further revealed *gpr137* modulated epithelial cell function and cell apoptosis. Men et al. (67) found that downregulation of *gpr137* expression promoted apoptosis. Combined with the upregulation of anti-apoptotic factors, it can be inferred that the high expression of *gpr137* may have slowed down apoptosis in the RL group.

**Figure 9:**
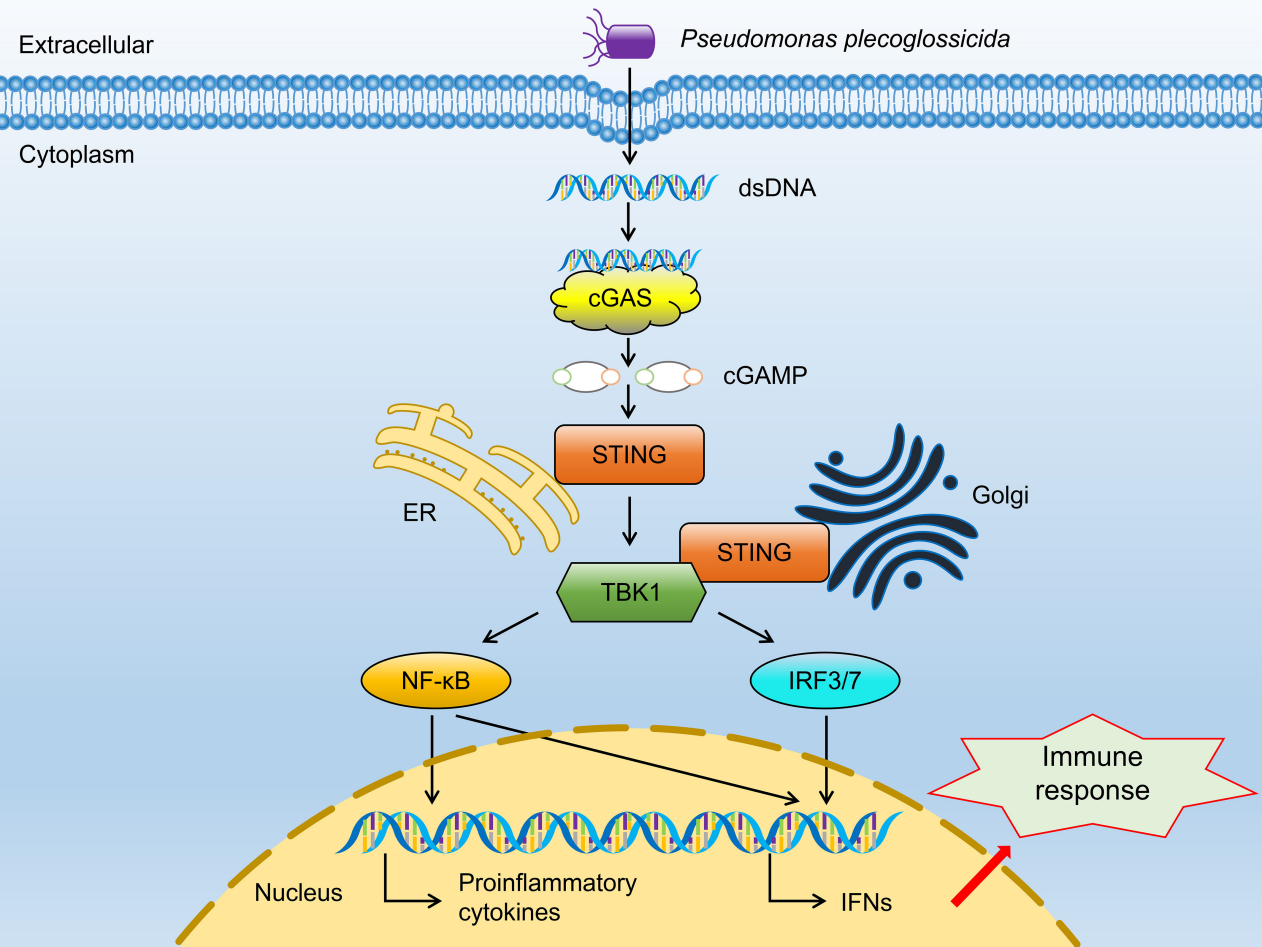
The regulatory network of the canonical cGAS-STING signaling pathway.

Alongside the three genes previously discussed, stimulator of interferon response cGAMP interactor 1 (*sting1*) emerges as a crucial upregulated gene in the RL group. Notably, DEGs related to VWND resistance show significant enrichment in the NOD-like receptor signaling pathway, which involves *sting1*, a vital component of innate immune and inflammatory responses (68). NOD-like receptor X1 negatively regulates the production of type-I IFNS by STING1 in innate immunity of host (69). Mechanistically, STING1 promotes NOD-like receptor protein 3 (NLRP3) activation to induce inflammation and pyroptosis (70). These studies show that the STING1-NLRs regulated network may be widely implicated in regulating the innate immunity of the host after pathogen infection. Nucleic acids of pathogens—important pathogen-associated molecular patterns—are one of the key factors in the activation of innate immune signaling pathways. The rapid recognition of cytosolic DNA by different pattern recognition receptors results in the induction of downstream anti-microbial effector genes (71). STING1 has been identified as a central adaptor in the innate immune response to cytosolic DNA (72). In the present study, the cytosolic DNA-sensing pathway was significantly enriched in the ST trait, indicating that the bacterial recognition pathway may be important for VWND resistance. Moreover, interferon-inducible protein 16 (a DNA sensing molecule) interacts with STING1 to promote it phosphorylation and translocation to mediate NF-κB signaling (73, 74). After recognizing cyclic dinucleotides of bacteria, STING1 recruits and activates TANK-binding kinase 1 (TBK1), which in turn phosphorylates interferon regulatory factor 3 (IRF3) and interferon regulatory factor 7 (IRF7), thereby resulting in the production of both type I IFNs and pro-inflammatory cytokines (75). In this work, *sting1* exhibited significantly higher expression levels in the RL group following VWND infection, suggesting that IFNs may assist the host defense against invading pathogens. Previous research showed that *tbk1*, *irf3*, and *irf7* were significantly upregulated post bacterial pathogen-associated molecular patterns (LPS) stimulation in *sting1* overexpressing cells of *Ctenopharyngodon idella* (76). Further research indicated that *in vivo sting1* over-expression decreases the sensitivity of *Oreochromis niloticus* to *Streptococcus agalactiae* infection (77). STING1^-/-^ mice exhibited reduced production of type I IFNs and excessive inflammatory response accompanied by decreased resistance to *Pseudomonas aeruginosa* challenge, resulting in significantly higher mortality (78). *Sting1* serves as a link between the cytosolic DNA-sensing pathway enriched in GAWS and the NOD-like receptor signaling pathway identified in the transcriptome analysis. Therefore, through recognizing double-stranded DNA (dsDNA) from *P. plecoglossicida* and inducing the transcription of pro-inflammatory cytokines and interferons (IFNS), the *sting1*-mediated cGAS-STING signaling pathway shows the importance for the resistance to VWND in *L. polyactis* (**Figure 9**). Several significantly upregulated genes compared to the CL and SL group were identified in the RL group, including *cgas*, *sting1*, *tbk1*, *irf3*, *irf7*, *nfκb2*, *ifnc*, *ifnd*, *ifnh*, *ifnγ*, *il-6*, *il-1β*, and *tnfα*, all of which are involved in the cGAS-STING1 signaling pathway. Similarly, Zuo et al. (79) revealed that immune genes encoding inflammatory cytokines (such as *il-1β*, *il-6*, *ifnγ*, and *tnfα*), acute phase reactants were expressed differently in the susceptible fish and survivors - resistant fish after *Yersinia ruckeri* infection. Besides, the protein expression level of STING1 was significantly higher in the RL group compared to both the CL and SL groups. These findings indicate that the activation of the cGAS-STING signaling pathway may prolong the survival time of *L. polyactis* post-infection. However, the results indicated that *nfκb1* and *p65* exhibited different expression patterns, suggesting the presence of a negative feedback regulatory mechanism (80). Further studies are needed to elucidate the specific functions and roles of this pathway. Furthermore, previous studies have shown that members of the NF-*κ*B and IRF families are cross-regulated during the innate immune response (81). This evidence suggests that the cGAS-STING1 signaling pathway may be closely related to the innate immune response against VWND infection. Moreover, extensive research has indicated that STING1 recognizes 2’3’-cGAMP, which is bound by cGAS and dsDNA, as well as the host’s own DNA (such as mitochondrial DNA) in the cytoplasm to initiate an IFN response (82, 83). Additionally, interferon-inducible protein 16 and cGAS cooperate in the activation of STING1 (70). Thus, these evidences suggest *sting1* may participate in host antibacterial responses by recognizing cyclic dinucleotides and activating immune cascades against VWND in *L. polyactis*. Although *sting1* has been extensively studied in the context of viral infections in aquatic animals (84, 85), its role in bacterial infection remains less explored; therefore, specific functions and roles need further investigation. In summary, the integration of transcriptomic response to infection and gene network analysis has enabled the identification of key biological process. Notably, there may be *sting1*-mediated cGAS-STING signaling pathway activation for the outcome of infection. Since its discovery, the protein regulated in development and DNA damage 1 (*ddit4l*) has garnered attention for its pivotal role in cellular responses to diverse stressors (86). In the present study, *ddit4l* stands out as the key gene significantly upregulated in the SL group. In *O. mykiss* macrophages, *ddit4l* expression is induced by LPS and zymosan (87). This gene functions to mediate inflammatory responses, cell death, and autophagy. Pastor et al. (88) observed that *ddit4l* expression increased in response to LPS in macrophages derived from bone marrow. They showed that loss of *ddit4l* protected the development of inflammation by decreasing the expression of proinflammatory cytokines. It has been reported that the stimulation of *ddit4l* expression in macrophages increases oxidized LDL-induced cell death (89). *Ddit4l* is a known negative regulator of mTOR signaling; its protein expression induces inhibition of mTOR signaling and promotes autophagy (90, 91). Considering the shorter survival time of the SL group, it can be speculated that ultra-high expression of *ddit4l* promotes excessive inflammation and autophagy, accelerates cell death, and increases susceptibility to VWND.

In contrast, *mtmr7* and *slc7a2*, which share a similar expression pattern, are significantly downregulated in the SL group. MTMR7 is known to represses proliferation in several cell types (92). And it is a phosphatase with links to prion disease susceptibility (93), as well as known to interact with MTMR complexes regulating autophagy and apoptosis (94). Thus, it can be suspected that *mtmr7* deficiency causes a disorder in programmed cell death. Meanwhile, SLC7A2, an inducible L-arginine transporter, plays a crucial role in innate and adaptive immunity in macrophages (95) and is implicated in wound repair (96). *Slc7a2* knockout blocked arginine transport and limited its activity after macrophage activation (97). In bacterially infected *slc7a2*-/- mice, the levels of proinflammatory cytokines (i.e., *g-csf*, *il-1β*, *il-1α*, and *tnf-α)* and chemokines (i.e., *cxcl1*, *ccl2*, *ccl3*, *ccl4*, *cxcl2*, and *ccl5*) were observed to decrease (98). Besides, Lee et al. (99) revealed *slc7a2* were involved in the induction of innate immune responses against mycobacterial infections through RNA-Seq analysis. Jiang et al. (100) demonstrated that reduced *slc7a2* expression correlated with fewer immune infiltrates. Therefore, low expression of *slc7a2* in the SL group may have contributed to an inadequate immune response to bacterial infection.

Considering the complex genetic architecture of VWND resistance trait, false-positive SNP associations are possible. Although *sting1* is a promising candidate gene, its definitive role in VWND resistance remains to be experimentally verified. Thus, functional validations *in vivo* and *in vitro* models, such as gene knockdown or overexpression studies, are necessary for subsequent experiments to conclusively establish its further role in conferring resistance.

## 5 Conclusion

In the present study, GWAS and transcriptome analyses were combined to disclose the genetic loci of resistance to VWND in *L. polyactis* and to provide novel insights into the underlying molecular mechanisms. GWAS identified 22 SNPs with significant suggestive association levels, most of which were located on Chromosome 4. And 60 candidate genes annotated from GWAS were enriched in DNA- sensing pathway. Comparative transcriptional analysis showed the activation of the *sting1*-mediated NOD-like receptor signaling pathway after *P. plecoglossicida* infection. Then, integration of GWAS and RNA-seq data identified 28 candidate genes were significantly associated with VWND resistance, and further identified seven key genes (i.e., *ddit4l, fam199x, mtmr7, slc7a2, gpr137, lonrf3*, and *sting1*). Moreover, qPCR and Western blot results showed that the mRNA level of genes related to cGAS-STING signaling pathway and protein level of STING1 were highly expressed in RL. These results collectively revealed that *sting1* may be a promising candidate gene for further functional studies on resistance to VWND. The findings of this study elucidate transcriptional regulatory mechanisms of host genetic resistance to VWND to support genetic selection and breeding; they also provide new biological insight for future research.

## Data Availability

The datasets presented in this study can be found in online repositories. The names of the repository/repositories and accession number(s) can be found in the article/**Supplementary Material**.
