# Supplementary material for: Genome-wide association study and transcriptomic analysis reveal the crucial role of sting1 in resistance to visceral white-nodules disease in Larimichthys polyactis
Source: Front Immunol. 2025 Apr 28;16:1562307. doi: 10.3389/fimmu.2025.1562307 (PMC12066304; doi:10.3389/fimmu.2025.1562307)
Supplement: Supplementary file 1 [file DataSheet1.pdf]

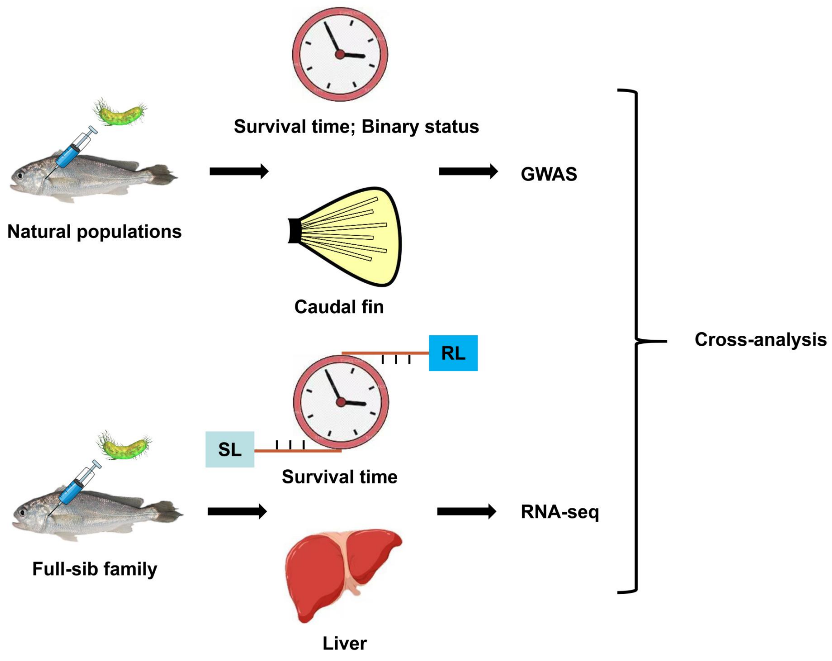

Fig. S1 A brief schematic diagram of the experimental.

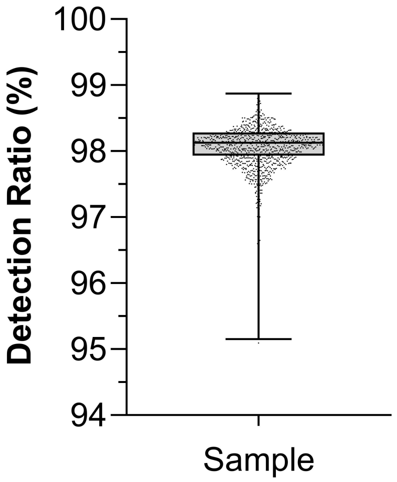

Fig. S2 The locus detection rate of samples.

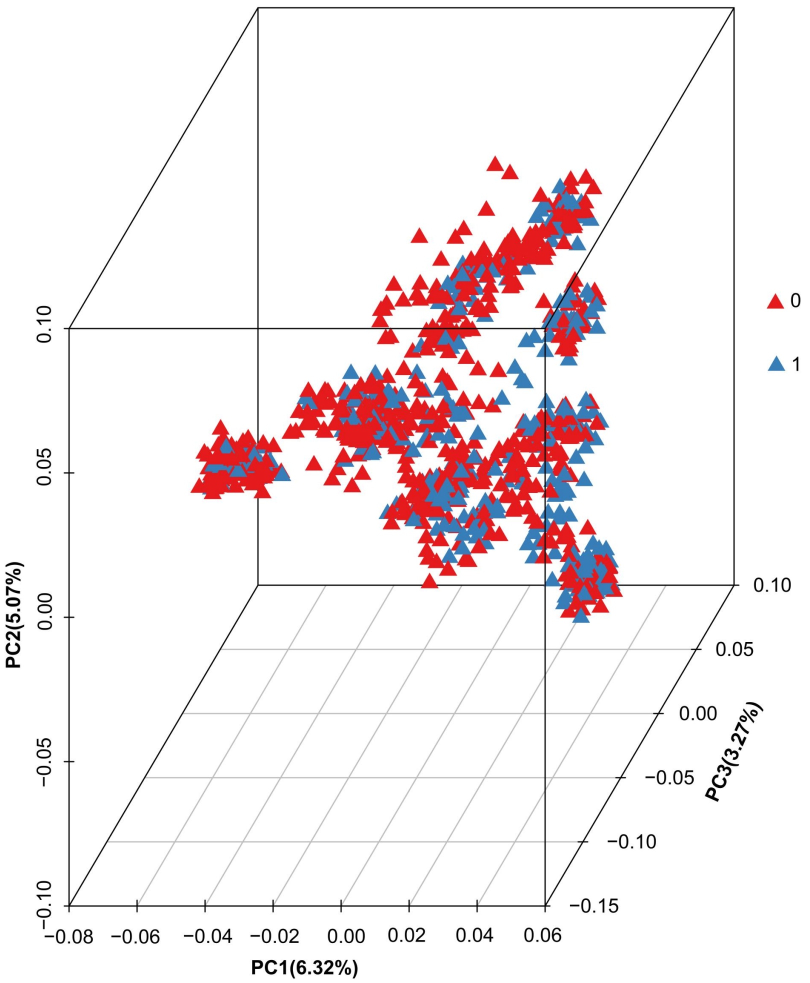

Fig. S3 Principal component analysis of population structure in *L. polyactis*. Red and blue triangles indicate 0 and 1 in binary status, respectively. The fish died before half of the total time to death was recorded as 0, and the rest fish was recorded as 1.

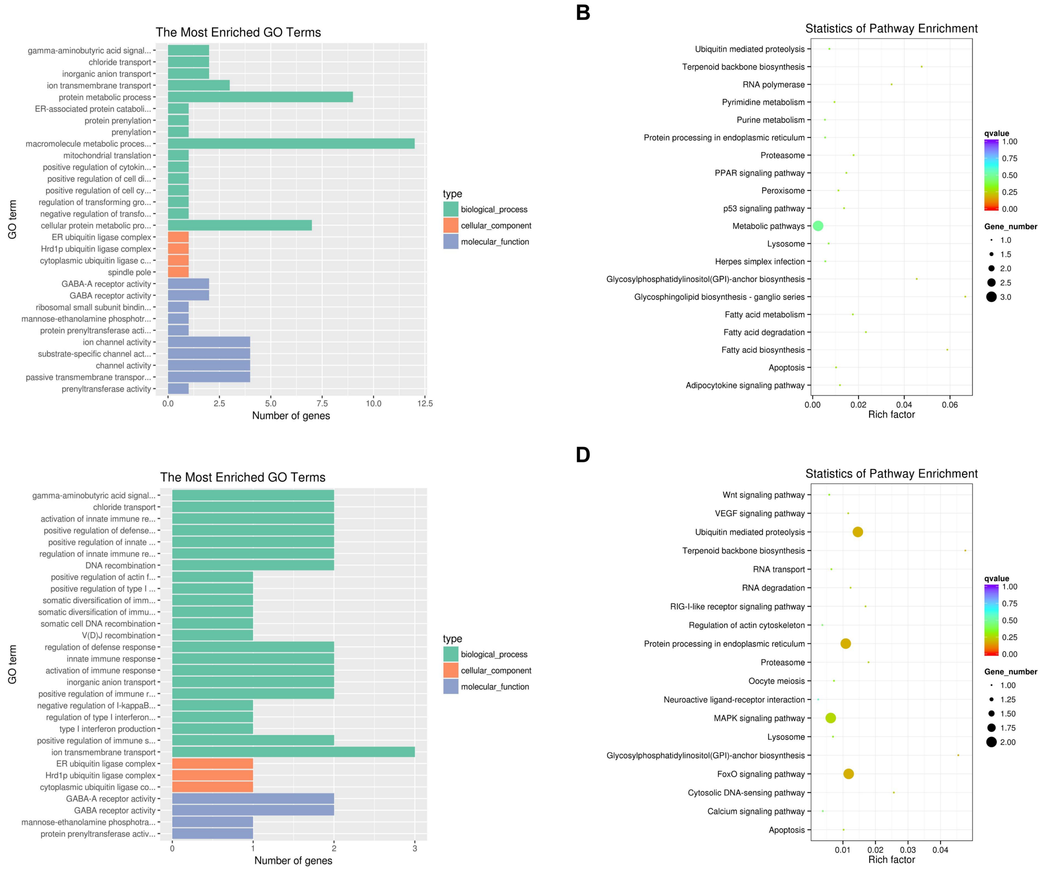

Fig. S4 Functional enrichment analysis of candidate genes in GWAS. (**A**) and (**C**) GO enrichment analysis of candidate genes in binary status and survival time, respectively. (**B**) and (**D**) KEGG enrichment analysis of candidate genes in binary status and survival time, respectively.

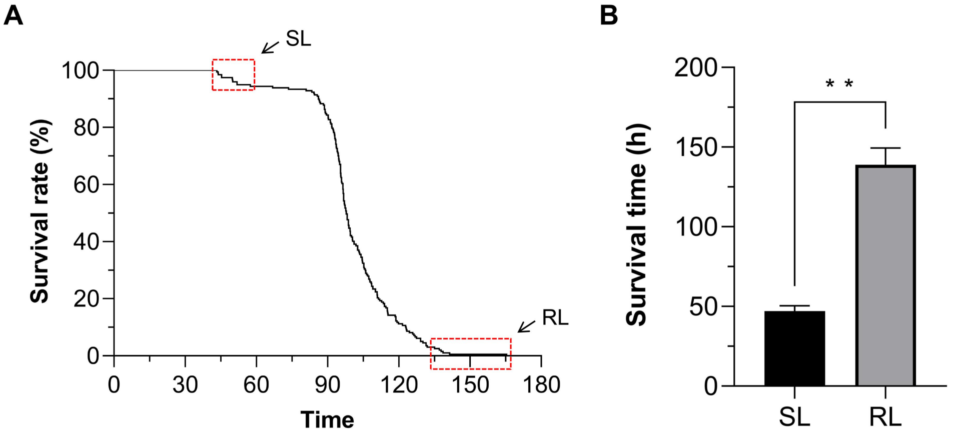

Fig. S5 (A) Survival curve of 200 *L. polyactis* after *P. plecoglossicida* infection. Red dotted line frames indicate the exact time frame of the sensitive fish (SL) and the resistant fish (RL),

respectively. The SL were sampled from 43.38 to 51.83 h post infection and the RL were sampled form 131.42 to 165.42 h post infection. (B) Difference of mean survival times between SL and RL groups. The mean survival times in SL and RL groups were 47.06 ± 3.10 h and 138.90 ± 9.95 h, respectively.

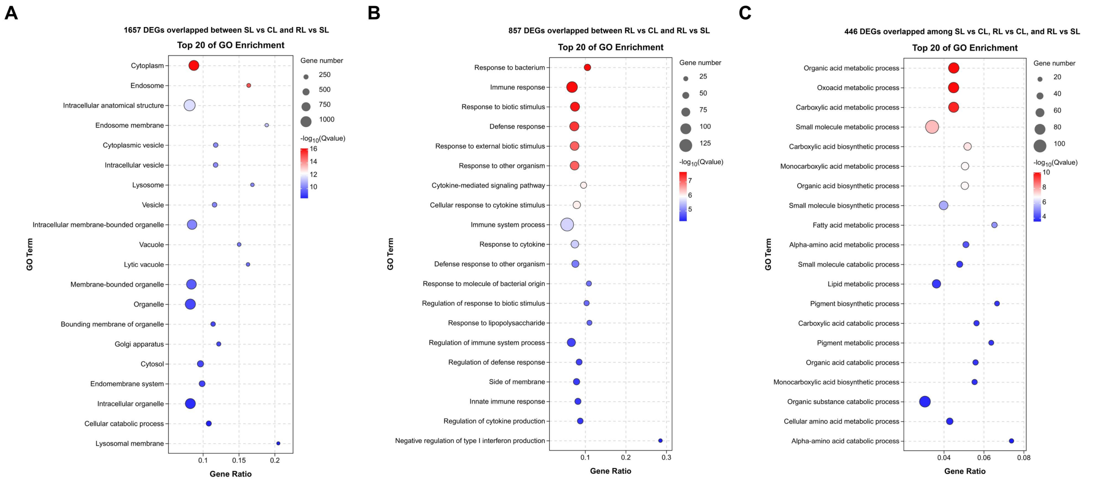

Fig. S6 Difference of transcriptomic expression patterns among CL, SL, and RL after infection. (**A**), (**B**) and (**C**) Bubble plot of the top 20 significantly enriched GO terms of 1657 DEGs overlapped between SL vs CL and RL vs SL, 857 DEGs overlapped between RL vs CL and RL vs SL and 466 DEGs overlapped among SL vs CL, RL vs CL, and RL vs SL, respectively.
