# Supplementary material for: Genome-wide association study and transcriptomic analysis reveal the crucial role of sting1 in resistance to visceral white-nodules disease in Larimichthys polyactis
Source: Front Immunol. 2025 Apr 28;16:1562307. doi: 10.3389/fimmu.2025.1562307 (PMC12066304; doi:10.3389/fimmu.2025.1562307)
Supplement: Supplementary file 2 [file DataSheet2.pdf]

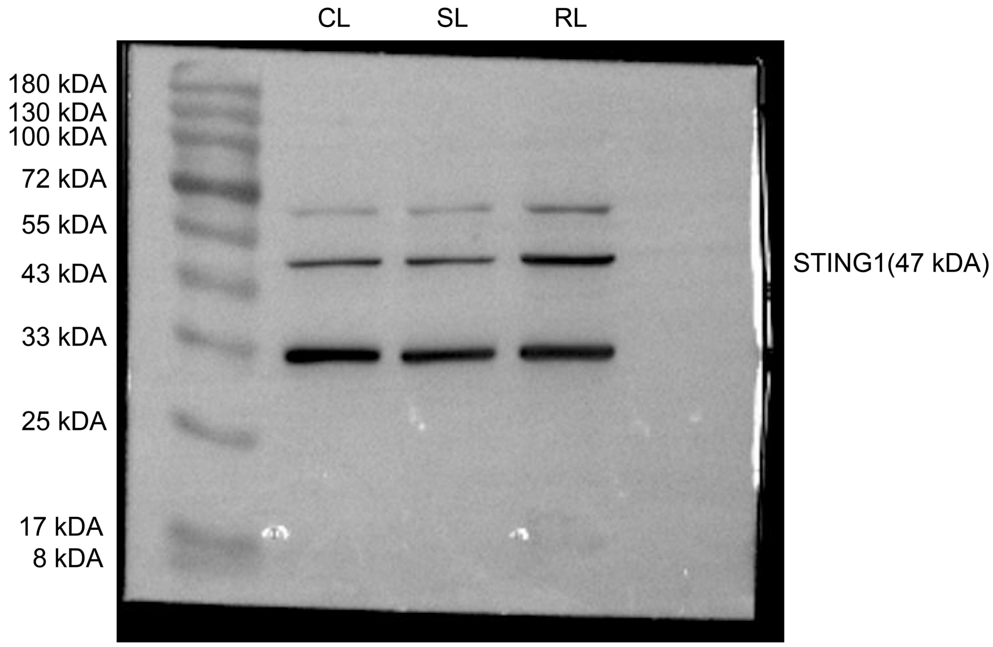

Fig. S1 Original Western blot image of STING1 (47 kDA).

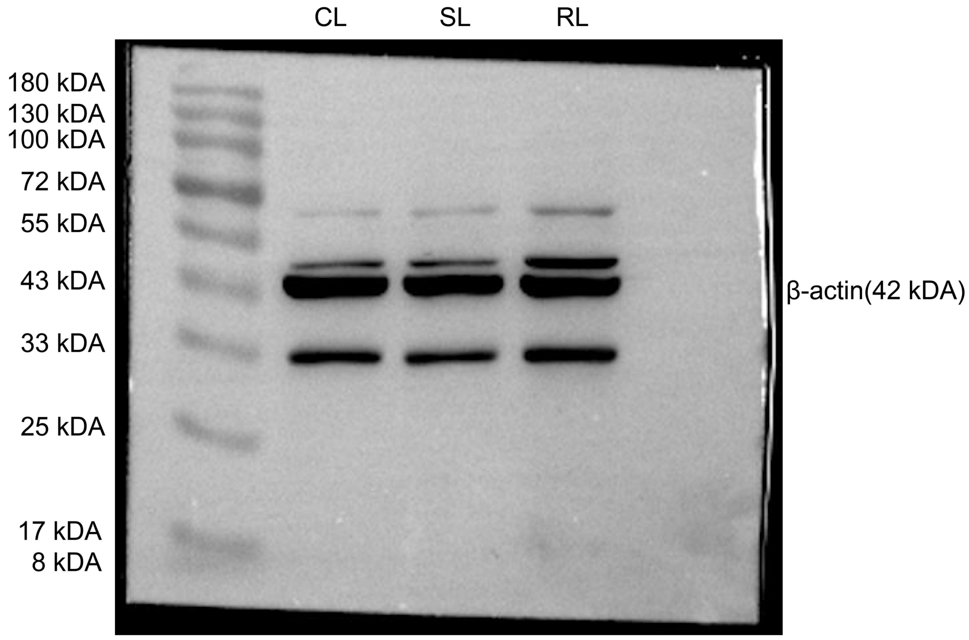

Fig. S2 Original Western blot image of β-actin (42 kDA).
