# Supplementary material for: Genome-wide association study and transcriptomic analysis reveal the crucial role of sting1 in resistance to visceral white-nodules disease in Larimichthys polyactis
Source: Front Immunol. 2025 Apr 28;16:1562307. doi: 10.3389/fimmu.2025.1562307 (PMC12066304; doi:10.3389/fimmu.2025.1562307)
Supplement: Supplementary file 3 [file Table1.doc]

| **Table S1** Primers used in this study | | |
| --- | --- | --- |
| Gene name | Forward sequences (5'-3') | Reverse sequences (5'-3') |
| *gpx3* | ATGTGGCCTGTTTTACCGCT | CTCCCGTTTAGAGTCCTGGC |
| *ube2d2* | GGAGTTGAACGACTTGGCAC | GAAGAAAACCCCGCTCTGGT |
| *tnip1* | CTGGTCACAGGAGGAGCAAC | CTTCGGTCTCACAGCTACCG |
| *rag1* | GCCCCTTTGCCTGACATTTG | CTCATCGTATCCCGTGCCTC |
| *rag2* | TGGACCACAACACATGGGAG | GAGCTTGACCAGCAAGGTCT |
| *zdhhc2* | GACGAACGGACTTCCAGACA | TCTGTTCTTGCAGACGAGCC |
| *ddit4l* | CAGATGACCGTCAGAGTGGG | TCAAACGTTGGGGTGACACT |
| *ctsf* | ATGGGACGTTGTTGTCCCTC | GACCGCCCAGCTTCTCAATA |
| *fgl1* | TGACAGACACCAACGCTACG | TTGATGTCCTGGTGACTGGC |
| *casp3l* | AAGATGGCAGAGGTTCGTGG | CGGTAAGGGTCCGATACTGC |
| *sting1* | GCTCCTCATCCTCATACCGC | ATAGCCCTTCCCATCCTCGT |
| *lcp1* | ACTCCAAGGCGTACTTCCAC | GCGTTTCCACACACGACATC |
| *syvn1* | TTGCCATTCGGCCCATGTAT | TTCAGGAGTAGCATCGGGGT |
| *tspan17* | TCTTCTTGGAGCTGACTGCG | TCGCGGTAAGCCTTGACATT |
| *snx25* | TACAGAGGAAGGTGGCGAGA | TGTGAGTTTCCTCTGGACGC |
| *st39a15* | TGATGAAGGAGACGACGCAC | CTCCCGTCAGGTCAGTGATG |
| *irf2* | GGATCCGAAGACGTGGAAGG | TCTGAAGGCGTTTGTTCCCT |
| *furin* | TCGTCAGTCACAGCGTTCTC | GCGTGAGGACAGAGGTACAC |
| *cnot7* | GGTCACTTGTACGGTCTGGG | TCACGACTGCTGCTTGTTGG |
| *cngc* | CAACGCATCCGATCCTGAGT | AATGGTGGTCAGCGTCAGAG |
| *gpr137* | GCCAACCACCTACCTGTTGT | GCCAGCCACAGTCTTTTGTC |
| *fam199x* | TGCCAAGTTCACCCTGTCTC | GTGCTGACCTTCCGACTCAG |
| *lonrf3* | ATGTTAAGGTGGAGGGCGTG | GCATGGGTCCAAAGTGTCCT |
| *mtmr7* | AGAAGTGTGTGAGCTGCGTT | TGCAACAGCCTTAGCGATGA |
| *slc7a2* | GGCCTTCATCACTGGTTGGA | CAGTATCAGGCAGACGGCAA |
| *β-actin* | CTCTGTCTGGATCGGAGGCT | GCTGAAGTTGTTGGGTGTTTG |

| **Table S2** Summary of 60 potential genes associated with visceral white-nodules disease resistance | | | | | | |
| --- | --- | --- | --- | --- | --- | --- |
| Trait | Chr | SNP Number | Gene | Start | End | Annotation |
| BS,  ST | 4 | 2 | *snx25* | 4911806 | 4924056 | Sorting Nexin-25 |
| BS,  ST | 4 | 2 | *cspp1* | 4928085 | 4937915 | Centrosome And Spindle Pole Associated Protein 1 |
| BS, ST | 4 | 5 | *map4k4* | 5419568 | 5505558 | Mitogen-activated protein kinase kinase kinase kinase 4 |
| BS, ST | 4 | 5 | *hdac3* | 5508593 | 5513992 | Histone Deacetylase 3 |
| BS, ST | 4 | 5 | *cfap100* | 5515674 | 5518919 | Cilia- And Flagella-Associated Protein 100 |
| BS, ST | 4 | 5 | *syvn1* | 5523617 | 5528818 | E3 Ubiquitin-Protein Ligase Synoviolin |
| BS, ST | 4 | 6 | *gpr137* | 5531282 | 5533869 | Integral Membrane Protein Gpr137 |
| BS, ST | 4 | 6 | *pld3* | 5537891 | 5546303 | Phospholipase D3 Isoform X1 |
| BS, ST | 4 | 6 | *smim19* | 5547778 | 5549111 | Small Integral Membrane Protein 19 |
| BS, ST | 4 | 6 | *nek1* | 5552485 | 5570650 | NIMA-related kinase 1 |
| BS, ST | 4 | 4 | *fam199x* | 5571350 | 5574956 | Family with sequence similarity 199, X-linked |
| BS, ST | 4 | 2 | *commd5* | 5577950 | 5581808 | Comm Domain-Containing Protein 5 |
| BS, ST | 4 | 2 | *lonrf3* | 5587269 | 5593550 | Lon Peptidase N-Terminal Domain And Ring Finger Protein 3 |
| BS, ST | 4 | 2 | *kiaa1211l* | 5604700 | 5641235 | Probable Serine/Threonine-Protein Kinase Kinx Isoform X2 |
| BS, ST | 4 | 2 | *aff2* | 5693478 | 5793426 | Af4/Fmr2 Family Member 2 |
| BS, ST | 4 | 2 | *mxra5* | 5852579 | 5882733 | Matrix-Remodeling-Associated Protein 5 |
| BS, ST | 4 | 2 | *ctsf* | 5907432 | 5912358 | Cathepsin F |
| BS, ST | 4 | 2 | *aup1* | 5913715 | 5921646 | Ancient Ubiquitous Protein 1 |
| BS, ST | 4 | 2 | *fnta* | 5924676 | 5928975 | Protein Farnesyltransferase/Geranylgeranyltransferase Type-1 Subunit Alpha |
| BS, ST | 4 | 2 | *tmem27* | 5938598 | 5939389 | Transmembrane Protein 271 |
| BS, ST | 4 | 3 | *pigg* | 5957472 | 6012681 | Gpi Gpi Ethanolamine Phosphate Transferase 2 |
| BS, ST | 4 | 1 | *guf1* | 6015946 | 6023743 | Translation Factor Guf1, Mitochondrial |
| BS, ST | 4 | 1 | *gabra2* | 6030093 | 6056461 | |Gamma-Aminobutyric Acid Receptor Subunit Alpha-2 |
| BS, ST | 4 | 1 | *psmd6* | 6037239 | 6038031 | 6S Proteasome Non-Atpase Regulatory Subunit 6 |
| BS, ST | 4 | 1 | *gabra4* | 6074226 | 6091254 | Gamma-Aminobutyric Acid Receptor Subunit Alpha-4 |
| BS | 4 | 1 | *irf2* | 4796305 | 4800854 | Interferon regulatory factor 2 |
| BS | 4 | 1 | *casp3l* | 4806782 | 4811616 | Caspase-3-Like |
| BS | 4 | 1 | *cenpu* | 4811978 | 4817608 | Centromere Protein U Isoform X1 |
| BS | 4 | 1 | *acsl1* | 4819437 | 4827136 | Long-Chain-Fatty-Acid--Coa Ligase 1 |
| BS | 4 | 1 | *st3gal5* | 4835968 | 4848300 | Lactosylceramide Alpha-2,3-Sialyltransferase Isoform X1 |
| BS | 4 | 1 | *polr1a* | 4851604 | 4868118 | Dna-Directed Rna Polymerase I Subunit Rpa1 |
| BS | 4 | 2 | *ptcd3* | 4868423 | 4875777 | Pentatricopeptide Repeat Domain-Containing Protein 3, Mitochondrial |
| BS | 4 | 1 | *lcp1* | 4882312 | 4890762 | Plastin-2 |
| BS | 4 | 1 | *cnga2* | 4894569 | 4902987 | Cyclic Nucleotide-Gated Cation Channel |
| BS | 4 | 1 | *fgl1* | 4905100 | 4907665 | Fibrinogen-Like Protein 1 |
| BS | 7 | 1 | *crygs* | 26581473 | 26581691 | Gamma-Crystallin B-Like |
| BS | 7 | 1 | *gs-1* | 26588463 | 26589194 | Gamma-Crystallin S-1 |
| BS | 7 | 2 | *crygb* | 26594779 | 26615160 | Gamma-Crystallin M3 |
| ST | 4 | 1 | *fmr1* | 5829677 | 5843301 | Synaptic Functional Regulator Fmr1 |
| ST | 4 | 1 | *ube2d2* | 6283155 | 6292413 | Ubiquitin-Conjugating Enzyme E2 D2 |
| ST | 4 | 1 | *ppp3ca* | 6300541 | 6332192 | Serine/Threonine-Protein Phosphatase 2B Catalytic Subunit Alpha Isoform-Like Isoform X1 |
| ST | 4 | 1 | *sting1* | 6337475 | 6344090 | Stimulator Of Interferon Genes Protein |
| ST | 4 | 1 | *ddit4l* | 6350872 | 6351850 | Dna Damage-Inducible Transcript 4-Like Protein |
| ST | 4 | 1 | *fgf20* | 6630310 | 6630711 | Fibroblast Growth Factor 20 |
| ST | 4 | 1 | *micu3* | 6632087 | 6649528 | Calcium Uptake Protein 3, Mitochondrial |
| ST | 4 | 1 | *zdhhc2* | 6652772 | 6662660 | Palmitoyltransferase Zdhhc2 |
| ST | 4 | 1 | *cnot7* | 6665660 | 6668841 | Ccr4-Not Transcription Complex Subunit 7 |
| ST | 4 | 1 | *mtmr7* | 6676540 | 6684514 | 7 Myotubularin-Related Protein 7 |
| ST | 4 | 1 | *slc7a2* | 6690647 | 6697156 | Cationic Amino Acid Transporter 2 Isoform X2 |
| ST | 4 | 1 | *pdgfrl* | 6700290 | 6705150 | Platelet-Derived Growth Factor Receptor-Like Protein |
| ST | 4 | 1 | *mtus1a* | 6707078 | 6718749 | Mitochondrial Tumor Suppressor 1 -Like Protein |
| ST | 4 | 1 | *tspan17* | 9169398 | 9186431 | Tetraspanin-17 |
| ST | 4 | 1 | *synpo* | 9191882 | 9202608 | Synaptopodin |
| ST | 4 | 1 | *dctn4* | 9234752 | 9242739 | Dynactin Subunit 4 Isoform X2 |
| ST | 4 | 1 | *gpx3* | 9245965 | 9263531 | Glutathione Peroxidase 3 |
| ST | 4 | 1 | *tnip1* | 9265482 | 9273277 | Tnfaip3-Interacting Protein 1 Isoform X5 |
| ST | 9 | 1 | *furin* | 14655879 | 14686796 | Furin |
| ST | 9 | 1 | *accs* | 14689760 | 14696336 | 1-Aminocyclopropane-1-Carboxylate Synthase-Like Protein 1 |
| ST | 9 | 1 | *rag1* | 14712976 | 14717131 | Recombination Activating Protein 1 |
| ST | 9 | 1 | *rag2* | 14719674 | 14721110 | Recombination Activating Protein 2 |

| **Table S3** Summary of RNA-seq reads and mapping statistics | | | |
| --- | --- | --- | --- |
| Reads summary | CL | SL | RL |
| Raw reads | 443,812,002 | 404,116,130 | 447,693,504 |
| Raw data / Gb | 66.57 | 60.62 | 67.15 |
| Clean reads | 440,624,088 | 401,078,390 | 443,937,350 |
| Clean data / Gb | 60.75 | 54.91 | 60.42 |
| GC content / % | 48.26 | 46.8 | 47.59 |
| Q20 | 97.89 | 97.98 | 97.84 |
| Total mapped reads  (ratios) | 397,001,093 (90.17%) | 350,009,348 (87.33%) | 394,847,034 (89.02%) |
| Unique mapped reads (ratios) | 367,758,937(83.55%) | 321,224,521 (80.13%) | 351,253,098 (79.20%) |

| **Table S4** Statistics for the Illumina sequencing and mapping of reads | | | | | | | | | | | |
| --- | --- | --- | --- | --- | --- | --- | --- | --- | --- | --- | --- |
| Group | Sample | Raw Reads | Raw Data(Gb) | Clean Reads(%) | Clean Data(Gb) | Q20(%) | Q30(%) | GC Data(Gb) | GC Content | Unique Mapped Reads(%) | Total Mapped Reads(%) |
| CL | CL_1 | 48,648,566 | 7.30 | 48,301,154(99.29%) | 6.63 | 6,489,080,963(97.93%) | 6,240,082,040(94.17%) | 3.13 | 47.24% | 40,024,390(82.99%) | 42,396,743(87.91%) |
|  | CL_2 | 47,720,716 | 7.16 | 47,363,086(99.25%) | 6.58 | 6,435,816,237(97.79%) | 6,180,965,094(93.91%) | 3.20 | 48.62% | 40,366,923(85.35%) | 43,206,595(91.35%) |
|  | CL_3 | 47,039,466 | 7.06 | 46,626,770(99.12%) | 6.45 | 6,306,117,562(97.78%) | 6,055,221,192(93.89%) | 3.11 | 48.15% | 38,672,278(83.01%) | 42,274,981(90.74%) |
|  | CL_4 | 49,113,190 | 7.37 | 48,733,210(99.23%) | 6.65 | 6,503,999,080(97.77%) | 6,236,234,080(93.74%) | 3.27 | 49.09% | 40,914,567(84.01%) | 44,677,213(91.74%) |
|  | CL_5 | 49,998,042 | 7.50 | 49,672,102(99.35%) | 6.94 | 6,784,546,437(97.76%) | 6,512,679,424(93.84%) | 3.41 | 49.08% | 42,019,908(84.66%) | 45,302,910(91.27%) |
|  | CL_6 | 49,139,806 | 7.37 | 48,708,988(99.12%) | 6.67 | 6,538,587,933(98.08%) | 6,297,561,129(94.46%) | 3.32 | 49.75% | 41,082,952(84.42%) | 44,903,592(92.27%) |
|  | CL_7 | 49,837,682 | 7.48 | 49,622,330(99.57%) | 6.94 | 6,792,868,340(97.93%) | 6,535,538,213(94.22%) | 3.26 | 47.01% | 40,306,785(81.38%) | 43,448,399(87.73%) |
|  | CL_8 | 58,943,680 | 8.84 | 58,477,574(99.21%) | 7.98 | 7,821,502,312(97.95%) | 7,526,576,787(94.26%) | 3.85 | 48.28% | 48,941,263(83.82%) | 53,066,968(90.88%) |
|  | CL_9 | 43,370,854 | 6.51 | 43,118,874(99.42%) | 5.91 | 5,797,289,216(98.02%) | 5,581,989,004(94.38%) | 2.79 | 47.10% | 35,429,871(82.30%) | 37,723,692(87.62%) |
| SL | SL_1 | 36,648,378 | 5.50 | 36,428,108(99.40%) | 5.06 | 4,957,210,218(97.92%) | 4,772,172,828(94.27%) | 2.36 | 46.67% | 29,360,263(80.74%) | 31,766,753(87.36%) |
|  | SL_2 | 59,722,726 | 8.96 | 59,185,116(99.10%) | 8.06 | 7,898,346,358(97.96%) | 7,601,237,336(94.28%) | 3.87 | 47.99% | 49,027,379(82.97%) | 53,182,184(90.00%) |
|  | SL_3 | 38,753,582 | 5.81 | 38,503,060(99.35%) | 5.32 | 5,220,157,582(98.14%) | 5,033,758,111(94.64%) | 2.48 | 46.71% | 30,786,743(80.27%) | 33,485,668(87.31%) |
|  | SL_4 | 37,587,884 | 5.64 | 37,373,344(99.43%) | 5.18 | 5,081,994,557(98.04%) | 4,895,369,053(94.44%) | 2.40 | 46.39% | 29,265,621(78.45%) | 31,990,013(85.75%) |
|  | SL_5 | 39,064,578 | 5.86 | 38,816,076(99.36%) | 5.38 | 5,278,752,420(98.16%) | 5,094,819,669(94.74%) | 2.53 | 47.02% | 30,888,072(79.69%) | 34,009,989(87.75%) |
|  | SL_6 | 48,923,802 | 7.34 | 48,449,698(99.03%) | 6.54 | 6,413,278,574(97.99%) | 6,176,490,445(94.37%) | 3.09 | 47.23% | 40,008,810(82.64%) | 43,767,706(90.40%) |
|  | SL_7 | 47,850,312 | 7.18 | 47,515,898(99.30%) | 6.53 | 6,390,919,109(97.81%) | 6,140,059,979(93.97%) | 3.03 | 46.43% | 37,981,763(80.07%) | 40,856,423(86.13%) |
|  | SL_8 | 47,938,546 | 7.19 | 47,596,272(99.29%) | 6.47 | 6,326,866,849(97.78%) | 6,075,811,639(93.90%) | 2.98 | 46.00% | 36,377,328(76.76%) | 39,297,453(82.92%) |
|  | SL_9 | 47,626,322 | 7.14 | 47,210,818(99.13%) | 6.35 | 6,229,605,049(98.05%) | 6,001,772,817(94.46%) | 2.97 | 46.75% | 37,528,542(79.61%) | 41,653,159(88.36%) |
| RL | RL_1 | 48,553,326 | 7.28 | 48,239,572(99.35%) | 6.71 | 6,574,713,737(98.01%) | 6,330,353,057(94.37%) | 3.20 | 47.64% | 38,934,404(80.77%) | 43,350,082(89.93%) |
|  | RL_2 | 48,495,970 | 7.27 | 48,035,484(99.05%) | 6.46 | 6,287,978,463(97.29%) | 5,997,787,439(92.80%) | 3.01 | 46.60% | 37,386,239(77.94%) | 41,964,362(87.48%) |
|  | RL_3 | 53,704,658 | 8.06 | 53,292,972(99.23%) | 7.23 | 7,097,008,445(98.10%) | 6,845,043,907(94.62%) | 3.40 | 47.06% | 42,185,257(79.24%) | 46,850,925(88.01%) |
|  | RL_4 | 47,439,440 | 7.12 | 47,023,110(99.12%) | 6.37 | 6,225,673,905(97.80%) | 5,982,165,793(93.98%) | 3.05 | 47.95% | 37,216,191(79.20%) | 42,117,195(89.63%) |
|  | RL_5 | 48,618,546 | 7.29 | 48,181,424(99.10%) | 6.54 | 6,396,074,780(97.84%) | 6,147,021,519(94.03%) | 3.19 | 48.83% | 38,317,608(79.59%) | 42,916,694(89.14%) |
|  | RL_6 | 56,919,480 | 8.54 | 56,449,628(99.17%) | 7.68 | 7,528,289,669(98.07%) | 7,254,138,082(94.50%) | 3.64 | 47.37% | 43,900,341(77.86%) | 49,932,926(88.56%) |
|  | RL_7 | 54,149,096 | 8.12 | 53,689,310(99.15%) | 7.33 | 7,177,054,586(97.85%) | 6,896,861,589(94.03%) | 3.49 | 47.63% | 43,135,814(80.40%) | 48,758,436(90.88%) |
|  | RL_8 | 47,325,692 | 7.10 | 46,896,854(99.09%) | 6.36 | 6,208,227,898(97.66%) | 5,951,113,208(93.61%) | 3.03 | 47.67% | 37,054,709(79.08%) | 41,708,592(89.01%) |
|  | RL_9 | 42,487,296 | 6.37 | 42,128,996(99.16%) | 5.74 | 5,627,440,887(97.96%) | 5,414,339,785(94.26%) | 2.73 | 47.57% | 33,122,535(78.75%) | 37,247,822(88.56%) |
